# A set of vectors and strains for chromosomal integration in fission yeast

**DOI:** 10.1038/s41598-023-36267-1

**Published:** 2023-06-08

**Authors:** Akihisa Matsuyama, Atsushi Hashimoto, Shinichi Nishimura, Minoru Yoshida

**Affiliations:** 1 Chemical Genomics Research Group, RIKEN Center for Sustainable Resource Science, 2-1, Hirosawa, Wako, Saitama 351-0198 Japan; 2 Laboratory of Microbiology, Department of Biotechnology, Graduate School of Agricultural and Life Sciences, The University of Tokyo, Tokyo, 113-8657 Japan; 3 Collaborative Research Institute for Innovative Microbiology, The University of Tokyo, Tokyo, 113-8657 Japan; 4 Graduate School of Integrated Sciences for Life, Hiroshima University, Higashi-Hiroshima, 739-8528 Japan

**Keywords:** Genetics, Microbiology, Molecular biology

## Abstract

The expression of heterologous genes is an important technique in yeast genetics. In fission yeast, the *leu1* and *ura4* genes have been used mainly as selectable markers for heterologous expression. To expand the repertoire of selection markers available for heterologous expression of genes, here we developed new host-vector systems employing *lys1* and *arg3*. By employing genome editing with the CRISPR/Cas9 system, we isolated several alleles of *lys1* and *arg3*, each having a critical mutation in the ORF region. In parallel, we developed a set of vectors that complement the amino acid auxotrophy of *lys1* and *arg3* mutants when integrated into each locus. Using these vectors in combination with the previously developed integration vector pDUAL, we successfully observed the localization of three proteins in a cell simultaneously by fusing them with different fluorescent proteins. Thus, these vectors enable combinatorial expression of heterologous genes, which addresses increasingly diverse experimental challenges.

## Introduction

The study of yeast biology has stimulated the development of sophisticated experimental systems for elucidating the molecular mechanisms underlying a variety of biological phenomena. Recently, to explore these molecular mechanisms more deeply, complicated experimental systems are sometimes required. In many cases, such a complicated experiment necessitates the expression of multiple transgenes in a cell. For this purpose, several vectors, which can be used as episomal plasmids or integrative vectors, and constitutive or inducible promoters have been developed^1, 2^. Expression systems that can reproduce conditions similar to physiological environments are preferred. In this sense, the use of integrative vectors is one of the leading-edge systems used in yeast biology rather than episomal vectors^3^.

**Figure 1:**
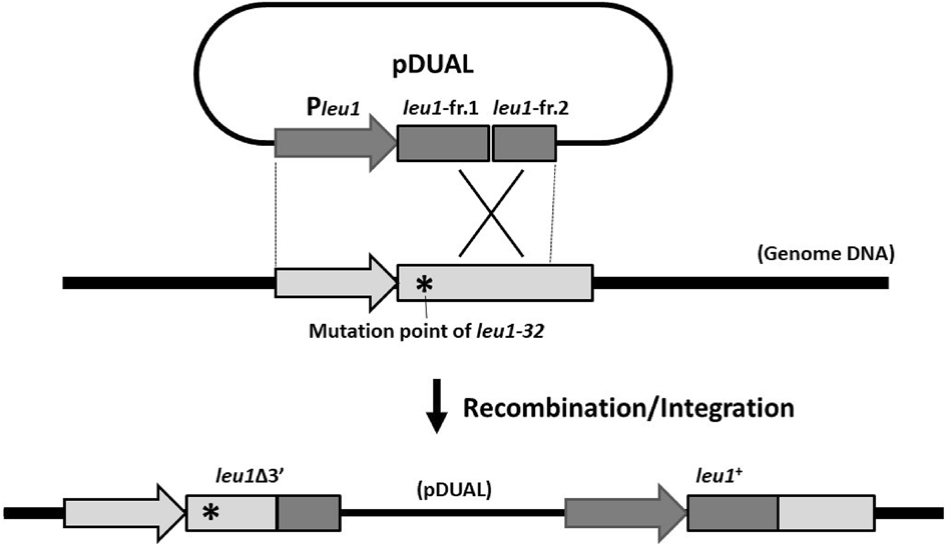
Integration of pDUAL into the genome of the *leu1-32* mutant. pDUAL series vectors have a part of the *leu1* gene containing restriction enzyme recognition sites within the *leu1* ORF region. When single crossover recombination occurs downstream of the mutation point of the *leu1-32* allele, the resultant genome is expected to have two copies of *leu1*, one which lacks the 3’ part and the other being a full-length functional gene. The schematic is not to scale.

We have previously developed two types of episomal vectors that can also be used for chromosomal integration by single crossover recombination^4, 5^. One type of vector is designed to be integrated into either the *lys1*, *arg1*, or *his3* loci. When these vectors are successfully integrated into the target loci, the transformants become auxotrophic for the corresponding amino acids. The merit of these vectors is that they can be used for strains having no amino acid auxotrophy. Therefore, in many cases, these vectors can be used without preparing a new host strain. However, the fact that the integration of the vectors into the genome results in amino acid auxotrophy is a disadvantage in some cases. On the other hand, the pDUAL series vectors are designed for integration into the *leu1* locus^4^. Although they can be used only for strains having the *leu1-32* allele, the transformants become prototrophic for leucine, enabling easy selection of the transformants (Fig. 1). The fact that the pDUAL vectors do not contain a functional copy of the *leu1* gene avoids the selection of transformants in which plasmids are randomly integrated into the genome. Although both types of vectors have their own advantages and disadvantages, pDUAL-type vectors seem more useful from the viewpoint of selection and maintenance of proper transformants.

In this paper, by taking the strategy of pDUAL-type single crossover recombination into account, we tried to develop new host-vector systems targeting loci other than *leu1*. Using the recently developed CRISPR/Cas9 system in fission yeast^6^, we successfully isolated strains with critical frameshift mutations in *lys1* or *arg3*^7, 8^. We also constructed vectors that could complement the amino acid auxotrophy of these mutants upon targeted integration into the chromosome. Together with the previously developed pDUAL vectors, expression of three transgenes in prototroph transformants is now available without using drug-resistant markers.

## Materials and methods

### *Schizosaccharomyces pombe* strains and media

*S. pombe* strains used in this study were derived from the wild-type strains JY743 (*h*^–^ *leu1-32 ura4-D18*) or AM324 (*h*^+^ *leu1-32*). The genomic DNA of wild-type JY3 (*h*^90^ prototroph) was used for PCR amplification of gene fragments for plasmid construction. Complete medium YE (0.5% yeast extract, 2% glucose, 5 µg/ml adenine) and minimal media SD and EMM2^9^ were used for culturing transformants. Leucine, arginine, lysine, histidine, and uracil were used at a final concentration of 50 µg/ml each, when needed. EMM2 was also used for transcriptional induction of ORFs fused with fluorescent proteins under the regulation of the *nmt1* promoter^10^.

### Genetic methods to handle *S*. *pombe*

Genetic methods to handle fission yeast were previously described^9^. Transformation of *S. pombe* cells was also described^11^. Ura^+^, Lys^+^, and Arg^+^ transformants were selected on SD medium lacking uracil, lysine, and arginine, respectively. When isolating mutant alleles of *lys1*, *arg1*, and *his1*, transformants were grown on SD medium containing leucine and either of lysine, arginine, or histidine, respectively.

### Isolation of auxotrophic mutant alleles of amino acid biosynthesis genes

Isolation of strains having critical mutations in genes involved in amino acid biosynthesis pathways was carried out using the CRISPR/Cas9 system as reported by Zhang et al*.*^12^, with slight modifications. Plasmids pDB4280 (cat. no. 98699) and pDB4282 (cat. no. 98701) for the fission yeast CRISPR/Cas9 system were provided from the Addgene repository. Design of single-guided RNAs (sgRNAs) was done using the web-based tool CRISPR4P^13^. Among target sites suggested by this program, we selected two sequences for each gene, which are positioned relatively close to the 5’ end of each ORF. Homology arms were shortened to 30 nt compared to the 40 nt indicated by Zhang et al.^12^. Sequences for annealing with the template vector pDB4282 were shortened to 20 nt, compared to the 23 nt indicated by Zhang et al. Primer sequences used were as follows: his1-sgRNA1 (TTGCCAAAAAACATAACCTGTACCGAAGAACAAGTCCAGGTACCTGAGTCGTTTTAGAGCTAGAAATAGC), his1-sgRNA2 (TTGCCAAAAAACATAACCTGTACCGAAGAAGGTGAGACAATGCGTGCATCGTTTTAGAGCTAGAAATAGC), arg3-sgRNA1 (TTGCCAAAAAACATAACCTGTACCGAAGAATCATGATCTCTCCGGTGACTGTTTTAGAGCTAGAAATAGC), arg3-sgRNA2 (TTGCCAAAAAACATAACCTGTACCGAAGAATCCATTCGTGATCTTAGTCGGTTTTAGAGCTAGAAATAGC), lys1-sgRNA1 (TTGCCAAAAAACATAACCTGTACCGAAGAATTTAGCCAAAGTGTGCGATCGTTTTAGAGCTAGAAATAGC), and lys1-sgRNA2 (TTGCCAAAAAACATAACCTGTACCGAAGAAATCCTCGTTAGTCGCATGACGTTTTAGAGCTAGAAATAGC). Each primer was used in PCR in combination with the ura4-P75 primer (CATCTGGTGTGTACAAAATTG) to amplify the region of pDB4282 containing a part of the *ura4* marker, the hammerhead ribozyme sequence, and the sgRNA scaffold sequence^12^. The PCR reaction was carried out in a 20 µl scale, and successful amplification was confirmed by agarose gel electrophoresis. The PCR product (2.5 µl) was directly used for transformation of the host strain JY743 together with the pDB4280 plasmid digested with NotI and purified by ethanol precipitation. One microgram of salmon sperm DNA was also used for transformation. Transformants were grown on an SD plate containing leucine and either histidine, arginine, or plate to prepare master plates. Amino acid auxotrophy of the transformants was then confirmed by streaking them on the SD plate lacking a corresponding amino acid.

All *lys1* and *arg3* mutants utilized in this study were deposited at the Yeast Genetic Resource Center (YGRC/NBRP) of Japan, alongside strains obtained from *lys1-K24* or *arg3-R25* mutants via genetic crosses with *ade6* and *ura4* mutants. The strains have been assigned accession IDs FY47712-FY47734.

### Sequence analysis of the mutant alleles

Sequence analysis of the lysine auxotrophic alleles was done as described below. The region around the sgRNA target site was amplified by PCR using the genome of each candidate. Primers used for amplification of the region around the target sites of lys1-sgRNA#1 and lys1-sgRNA#2 were lys1-F-puc119 (CTCTAGAGGATCCCCTTGACACTCTCCGTTAC) and lys1-R-puc119 (TCGAGCTCGGTACCCCACCGATAGGACCTGTAA). The resultant PCR fragments were cloned into the vector pUC119 by the gap-repair cloning technique^14^. The whole inserts were sequenced using a 3730xl DNA Analyzer (Applied Biosystems).

Sequence analysis was also done for mutant alleles of *his1* and *arg3* in a similar way. Primers used were as follows: arg3-F-puc119 (CTCTAGAGGATCCCCGCAGTCTGAGAGAGAACTAG) and arg3-R-puc119 (TCGAGCTCGGTACCCGTCTTTAGAAGCCTGTGTC) for *arg3*; and his1-F-puc119 (CTCTAGAGGATCCCCTGCATAGAGGGTCGTTT) and his1-R-puc119 (TCGAGCTCGGTACCCCGAGTGAATGCTGAGGA) for *his1*. PCR products were cloned into the *E*. *coli* vector pUC119 digested with SmaI.

### Vector construction

To construct the vector pCLys1, we replaced the *leu1* fragment of pDUAL containing the *nmt1* promoter^4^ with a part of the *lys1* gene containing the *lys1* promoter and the 5’ part of its ORF. This region was amplified by PCR using the genomic DNA of the wild-type *S*. *pombe* strain JY3. The restriction enzyme recognition site for NotI was generated within this region by catenating two PCR fragments containing the NotI site on one side by PCR. Primers used were as follows: KpnI-Plys1-F3 (GGGATAACAGGGTAATATGGTACCGAGCTGTTTGGACATGTTATG) and NotI-lys1-R3 (AACTACATCAGCGGCCGCTCTCAATTCCATTCTTTACGAG); and NotI-lys1-F4 (GGAATTGAGAGCGGCCGCTGATGTAGTTATGGTTTATGC) and EcoRI-lys1-R4 (CGTTGTAAAACGACGGCCAGTGAATTCGTCAGCTCCTTTTGAGACTG). Cloning of the catenated PCR fragment into the vector was done using the In-Fusion HD Kit (TaKaRa Bio).

Construction of the pCArg3 vector was carried out in a similar way. Primers used were as follows: KpnI-Parg3-F1 (GGGATAACAGGGTAATATACTAGTCGGTACCTGAGTTATCAATATAGTAACAC), NotI-arg3-R1 (AACGTTATTGCGGCCGCATCACCTACCCATGCAAC), NotI-arg3-F2 (GTAGGTGATGCGGCCGCAATAACGTTTTACATGATTTG), and EcoRI-arg3-R2 (TGTAAAACGACGGCCAGTGAATTCAGCATTTTTAACAGCAACC).

The full sequences of pCLys1 and pCArg3 are deposited in the DDBJ database with accession numbers LC745737 and LC745738, respectively. These plasmids were also deposited at the Yeast Genetic Resource Center of Japan with accession numbers for FYP5995 (pCLys1) and FYP5996 (pCArg3), so that they are available upon request.

Construction of pCLys1 and pCArg3 vectors containing GFP or mCherry was completed as described below. First, the ORF of GFP or mCherry was cloned into each of pCLys1 and pCArg3. Primers used for amplification of GFP were as follows: NheI-GFP-F (TTATAGTCGCTTTGTTAAAGCTAGCCTCGAGCCCGGGATGAGTAAAGGAGAAGAAC) and BamHI-GFP-R (GCTTATTTAGAAGTGGCGCGCCGGATCCCGGGTTTGTATAGTTCATCCATGCC). pDUAL-GFH1^4^ was used as a template for PCR. A GFP fragment was amplified and then mixed with pCLys1 or pCArg3 digested with NheI and BamHI, and the mixture was introduced into competent cells of the *E. coli* strain DH5α. mCherry was also cloned into the same vectors in a similar way. Primers used for amplification of mCherry were SmaI-mC-F (TTATAGTCGCTTTGTTAAAGCTAGCCTCGAGATGGTGAGCAAGGG) and SmaI-mC-R (ATTTAGAAGTGGCGCGCCGGATCCCGAGCTCGTCCATGCCGCCG). The amplified mCherry fragment was mixed with the vectors digested with SmaI, followed by introduction into *E. coli*.

ORFs to be fused with GFP or mCherry were amplified by PCR from our entry clone collection of the fission yeast ORFeome^15^. Primers used for amplification were Ent-GFP-R (CCTTTACTCATCCCGGGCTCGAGGCTAGCAACTTTGTACAAGAAAGCTGGGTA) for GFP-fusion and Ent-mC-R2 (CGCCCTTGCTCACCATCTCGAGGCTAGCAATGCCAACTTTGTACAAGAAAGCTG) for mCherry fusion, in combination with 221_pREY_F (ACTTATAGTCGCTTTGTTAAAGCTAGCGATATCAAAAAAGCAGGCTCTCATATG) as a partner of the above two primers. Each amplified ORF was cloned into pCLys1 or pCArg3 containing GFP or mCherry via gap-repair techniques. Cloning of an ORF into the pDUAL vector having YFP (pDUAL-YFH1c) was done by Gateway technology.

### Fluorescent microscopy

Fluorescence of CFP, GFP, YFP, and mCherry was observed using the all-in-one fluorescence microscope BZ-X700 (KEYENCE). The objective lens CFI Plan Apo λ 100XH (Nikon) was used. Cells were precultured on a YE or EMM2 plate containing thiamine and then streaked on an EMM2 plate to induce expression of fluorescent protein-fused proteins driven by the *nmt1* promoter.

## Results

### Isolation of amino acid auxotrophic mutants using the CRISPR/Cas9 system

To allow pDUAL-type single crossover chromosomal integration, strains having a point mutation in genes preferably encoding proteins functioning in the amino acid or nucleotide biosynthesis pathway are needed. There are several such auxotrophic mutants in fission yeast. However, the mutation points of most of them have not been identified or are not suitable for integration mediated complementation in view of the position. We therefore employed the CRISPR/Cas9 system to isolate such biosynthesis pathway mutants, according to the cloning-free method recently reported by Zhang et al.^12^.

In the field of fission yeast genetics, *leu1*, *ade6,* and *ura4* have been widely used as selection markers^16–18^. The *leu1* gene is located on the right arm of chromosome 2, whereas *ade6* and *ura4* are located on chromosome 3. We selected integration loci from chromosome 1 to enable easy handling in the genetic cross between new markers and the above three loci. As a result, we selected three genes—*lys1*, *arg3,* and *his1*^19^*—*as target loci. We then designed single-guided RNA sequences targeting the open reading frame of these genes, using the web-based sgRNA prediction program CRISPR4P^13^. Among the sgRNA target sequences suggested by CRISPR4P, we selected two sequences that are in the proximity of the initiation codon of each ORF, because an allele in which a point mutation is located close to either the 5’ or 3’ end of an ORF is suitable for single crossover chromosomal integration.

**Figure 2:**
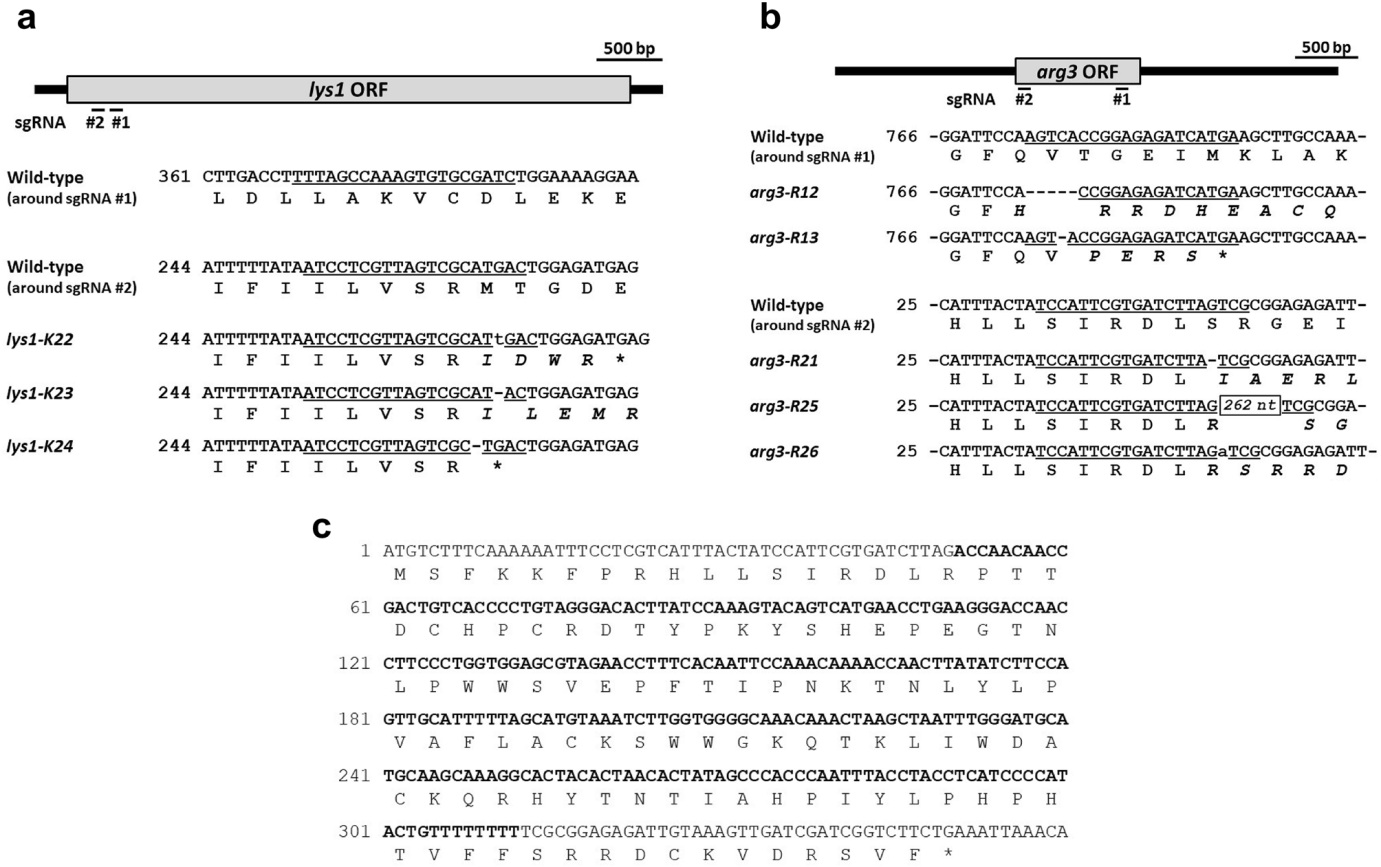
Mutations of auxotrophic mutants isolated in this study. All mutations of (**a**) lysine- and (**b**) arginine-auxotrophic mutants were found in the regions targeted by sgRNAs. Nucleotide sequences around the sgRNA target sequences are shown and amino acid sequences are shown beneath in the context of the reading frame. The number of the first nucleotide of each ORF is set to 1. The targeted sequences of sgRNA are underlined. An inserted nucleotide is shown in lowercase. A hyphen indicates a deleted nucleotide. An asterisk indicates a termination codon. Amino acid changes to the default sequence are indicated in italics. (**c**) The nucleotide and amino acid sequences of the *arg3-R25* allele. The nucleotide sequence inserted is shown in bold. The expected amino acid sequence of the protein expressed from the *arg3-R25* allele is shown below the nucleotide sequence.

We introduced PCR fragments including sgRNA target sequences and part of *ura4*^+^ into an *ura4-D18* mutant JY743 together with digested pDB4280 containing Cas9 and the 5’ part of *ura4*^+^^12^. After detecting Ura^+^ transformants, we checked the amino acid auxotrophy of the transformants. Similar to the situation observed for *his1*, many Ura^+^ transformants were obtained from both sgRNA #1 and #2 transformation. However, only a few strains showed amino acid auxotrophy, suggesting that these sgRNAs were not effective for targeting each site. The same was true for *lys1* sgRNA #1 transformants. On the contrary, in the case of *lys1* sgRNA #2 transformants, many transformants showed amino acid auxotrophy, despite the lower number of transformants compared with *lys1* sgRNA #1. Many auxotrophic transformants were also obtained from two different sgRNAs in the case of *arg3*. We subsequently analyzed whether and where a mutation has occurred in the targeted gene by sequencing after PCR amplification of the ORF region. As a result, we found several mutations in *lys1* and *arg3*, but not in *his1* (Fig. 2a,b). There were also several arginine auxotrophic mutants that had no mutations in the ORF region of *arg3*, similar to the situation of *his1*. This may be due to off-target effects, as is often discussed with the CRISPR/Cas9 system. Most mutations found were single nucleotide deletions or insertions, all of which occurred only in the sequence used as an sgRNA target.

Among these mutants, the *arg3-R25* allele contains an insert of 262 nucleotides (Fig. 2c). Consistent with this result, a longer product was obtained by PCR amplification of this locus when the genome of the *arg3-R25* strain was used as a template. A BLASTN search of this sequence revealed a complete match with a part of the genome of *Oncorhynchus tshawytscha*, known as chinook salmon or king salmon. This result strongly suggests that the 262-bp sequence came from the salmon sperm DNA used in the transformation of yeast cells, as previously reported^20^. Although the salmon sperm DNA we used originated from the salmon species *Oncorhynchus keta*, we could not find any sequence that matches the 262-bp sequence in the available *O. keta* dataset. Therefore, we have registered this sequence in the DDBJ database as part of the *O. keta* genome (accession no. LC764838). Because insertion of a long fragment was advantageous for chromosomal integration of a plasmid to avoid gene conversion-based complementation of a mutation^21^, we selected the *arg3-R25* allele that serves as a target for the integration vector. In the case of *lys1*, we selected the *lys1-K24* allele, having a deletion of adenine at the 268th nucleotide from the beginning of the *lys1* ORF, resulting in the generation of a termination codon just downstream of this mutation site (Fig. 2a).

### Construction of integration vectors

**Figure 3:**
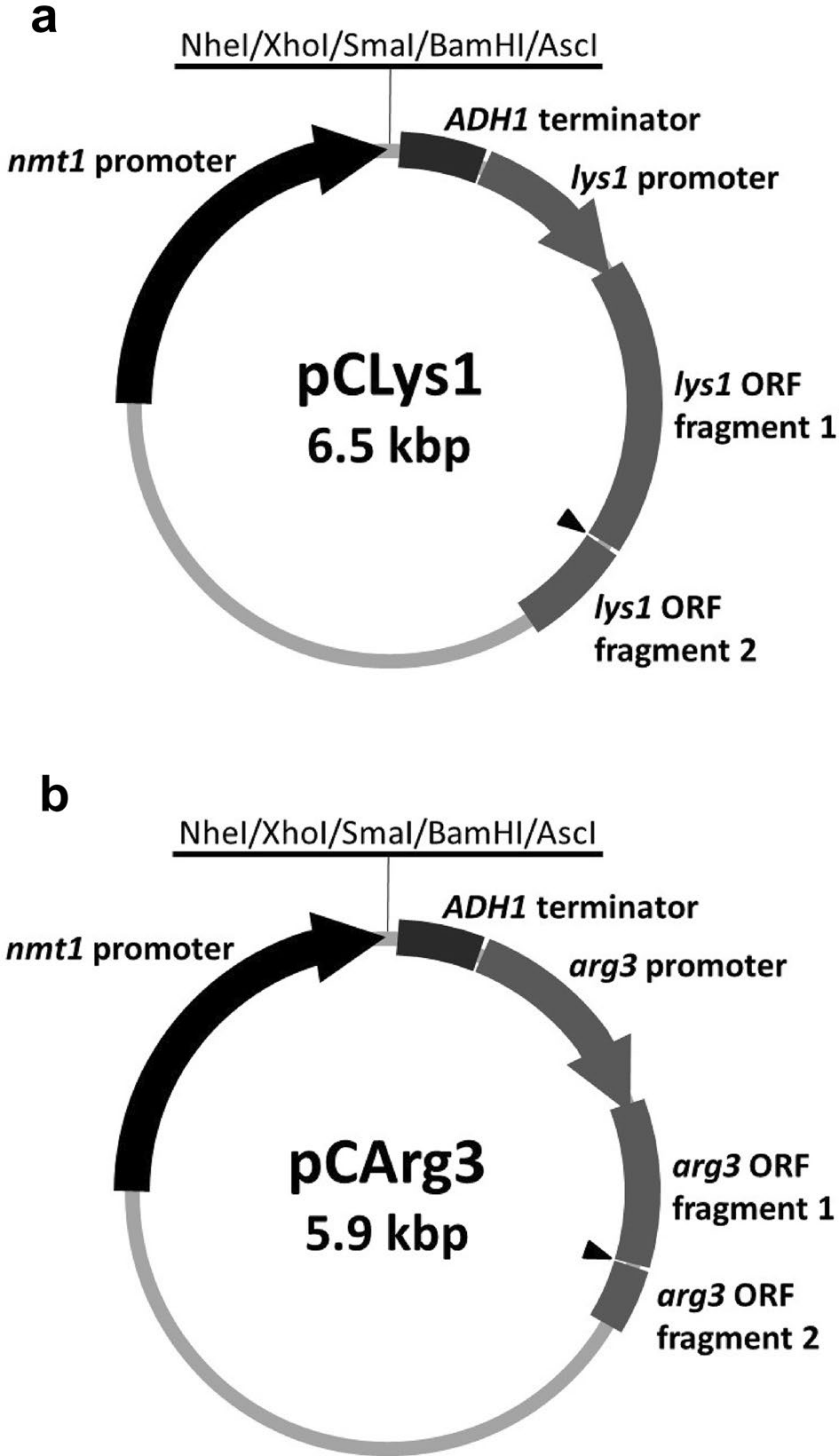
Vectors developed in this study. Structure of pCLys1 (**a**) and pCArg3 (**b**). The selection marker consists of the promoter and a part of the ORF of either *lys1* (pCLys1) or *arg3* (pCArg3). Both vectors have the *nmt1* promoter and the *ADH1* terminator for heterologous gene expression. Multicloning sites contain recognition sequences for NheI, XhoI, SmaI, BamHI, and AscI. An arrowhead indicates the NotI site for linearization of the plasmids. The schematic is not to scale.

**Figure 4:**
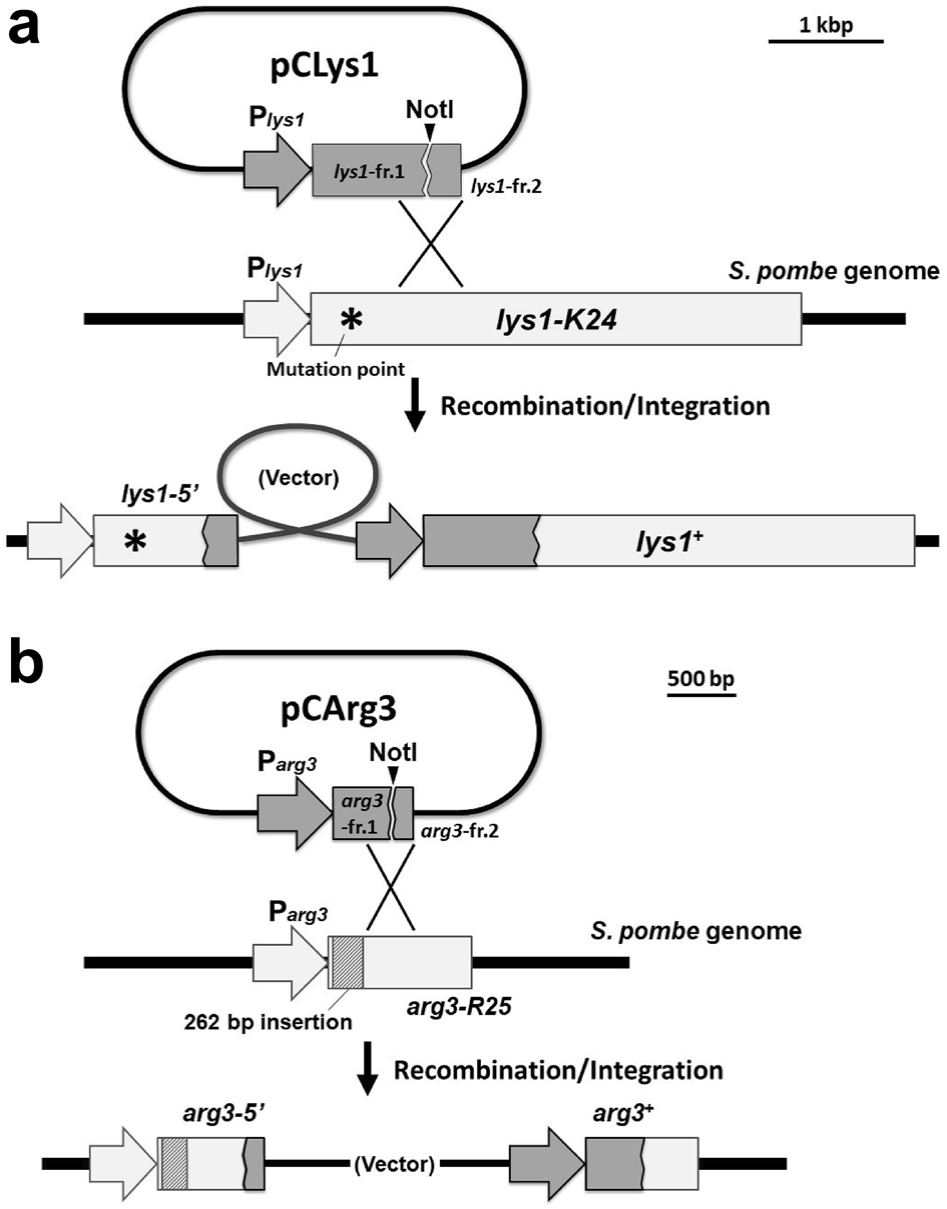
A conceptual diagram that summarizes integration of vectors into the genome. Integration of pCLys1 (**a**) and pCArg3 (**b**) into the chromosome is shown. An asterisk indicates the mutation point of the *lys1-K24* allele. A box with diagonal lines indicates a 262 bp fragment inserted within the ORF of *arg3*. The length of the vector backbone is not to scale.

In parallel with mutant isolation, we designed and constructed vectors for chromosomal integration. For the construction of a vector named pCLys1 (Fig. 3a) that can be integrated into the *lys1* locus, we amplified the genomic region containing the promoter and 5’ part of the *lys1* ORF and replaced the *leu1* region of the pDUAL vector with them. The cloned *lys1* fragment was expected to be non-functional, because only less than 30% of the *lys1* ORF was cloned. This short *lys1* fragment was first amplified by PCR from the wild-type genome as two parts and then fused with a NotI recognition site between them. Thus, the cloned partial gene could be split into two in the ORF region by NotI digestion. The NotI site was created approximately 700 bp downstream of the mutation point of the *lys1-K24* allele. If the pCLys1 vector linearized by NotI digestion is integrated into the *lys1* locus having the *lys1-K24* mutation, a functional *lys1* gene would be generated, and the transformants are expected to be lysine prototrophic (Fig. 4a).

We also constructed the pCArg3 vector for integration into the *arg3* locus through a similar process (Fig. 3b). As was the case for *lys1*, the partial *arg3* fragment cloned into the pCArg3 vector was expected to be non-functional, because the end of the cloned fragment was still upstream of the mutation points in the alleles obtained by *arg3* sgRNA #1 transformation.

### Chromosomal integration using the newly developed vectors

We subsequently examined whether the newly developed vectors could be integrated into the target loci as expected (Fig. 4a,b). If strains having the *lys1-K24* or *arg3-R25* alleles are transformed with pCLys1 or pCArg3, respectively, prototrophic transformants would be obtained. We linearized these plasmids with NotI prior to the transformation of the yeast cells. As a control, we introduced the plasmids directly into the auxotrophic mutants to examine whether linearization is required for integration or not.

**Figure 5:**
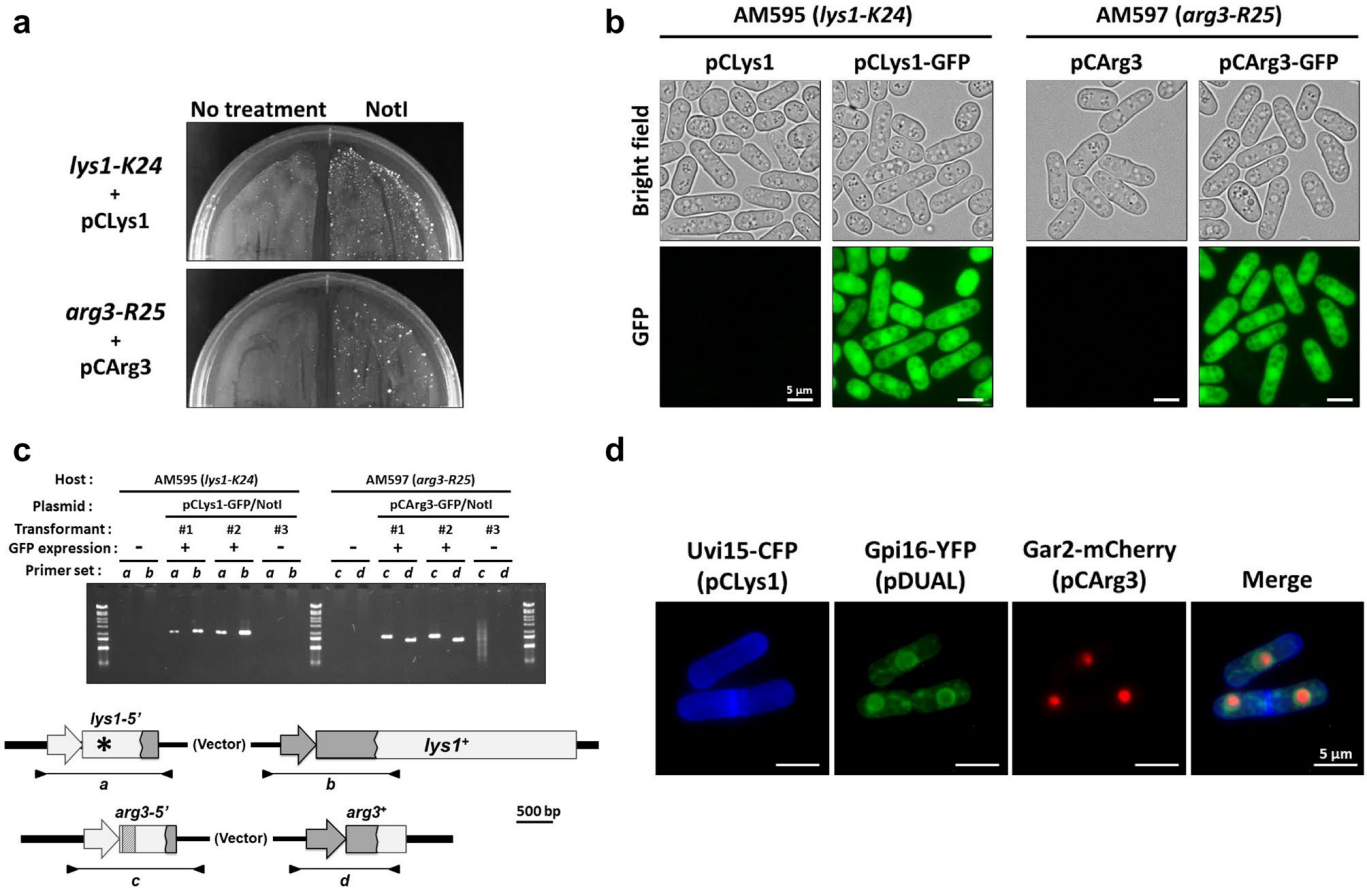
Introduction of newly developed vectors for heterologous gene expression. (**a**) Transformants that emerged after introduction of linearized plasmids developed in this study. Strains AM595 (*h*^–^ *leu1-32 lys1-K24*) and AM597 (*h*^–^ *leu1-32 arg3-R25*) were transformed with either pCLys1 or pCArg3 with or without NotI pretreatment and plated onto SD selection medium lacking lysine and arginine. (**b**) Expression of GFP introduced using pCLys1 and pCArg3. Transformants that emerged after introduction of linearized pCLys1-GFP and pCArg3-GFP into the *lys1-K24* or *arg3-R25* strains were detected and GFP fluorescence was monitored under fluorescence microscopy. (**c**) Confirmation of chromosomal integration by PCR. Integration of pCLys1-GFP or pCArg3-GFP into the chromosome was analyzed by PCR using primers indicated by arrowheads. Transformants #1 and #2 were strains in which GFP fluorescence was observed, whereas transformant #3 had no fluorescence. Lambda DNA digested with EcoT14I was used as a size marker. (**d**) Simultaneous expression of three proteins in a cell. Cells transformed with pCLys1, pCArg3, and pDUAL expressing *uvi15*-*CFP*, *gpi16*-*YFP*, and *gar2*-*mCherry*, respectively, were observed under fluorescence microscopy. Scale bars indicate 10 μm.

After the introduction of plasmids or their digested fragments into auxotrophic mutants, large colonies appeared only from the strains transformed with digested fragments for both *lys1-K24* and *arg3-R25* (Fig. 5a). Although tiny colonies appeared from the strains transformed with undigested plasmids, they did not become enlarged. To see whether any given gene could be expressed using the vectors developed in this study, we cloned an ORF encoding green fluorescent protein (GFP) into pCLys1 and pCArg3 as a representative. We integrated these plasmids into the chromosome and picked up some transformants without amino acid auxotrophy. When we checked GFP fluorescence after streaking transformants on an EMM2 plate, we observed signals that dispersed throughout the cells with a slight accumulation in the nucleus, consistent with the localization of free GFP proteins in fission yeast (Fig. 5b)^22^. To confirm that digested fragments were successfully integrated into the target loci, we examined the genome structure of the prototrophic transformants by colony PCR. This analysis demonstrated that all of the fragments derived from pCLys1-GFP or pCArg3-GFP were integrated into each target locus as expected only when GFP fluorescence was detected (Fig. 5c).

### Simultaneous expression of three genes using integration vectors

Finally, we examined whether 3 genes could be simultaneously expressed using the vectors developed in this study in combination with the previously developed pDUAL vector. To detect their expression, we employed three fluorescent proteins, CFP, YFP, and mCherry. We identified several proteins showing clear subcellular localization from our localization data archive^15^. We selected the nucleolar protein Gar2, the ER protein Gpi16, and Uvi15, which localizes at cell tips and septa, and cloned their ORFs upstream of CFP (pCLys1), YFP (pDUAL), and mCherry (pCArg3), respectively. These fusion genes were expressed under the control of the *nmt1* promoter. We integrated these plasmids sequentially into the chromosome of a strain with the *arg3-R25*, *lys1-K24,* and *leu1-32* alleles, and picked up some transformants exhibiting no amino acid auxotrophy. We also attempted to transform cells with all three plasmid fragments. However, we were unable to obtain any transformants under these conditions. Additionally, even when two out of the three plasmids were used, the number of transformants obtained was nearly negligible. When we checked the fluorescence after cultivating transformants on an EMM2 plate to activate the *nmt1* promoter, we observed clear signals from all fusion proteins (Fig. 5d). As expected, Uvi15 showed constant localization to cell tips or septa, whereas Gpi16 was visualized at ER-like membranous structures. A strong signal of the nucleolar protein Gar2 was observed in a specific compartment in the nucleus. Thus, we demonstrated that the introduction and expression of three different transgenes is possible using the newly developed integration vectors.

## Discussion

We developed two sets of fission yeast host-vector systems that could be used for heterologous expression of genes from either the *lys1* or *arg3* loci. Using these vectors, we demonstrated that developed vectors were properly integrated into the target loci, resulting in the generation of prototroph transformants. In addition, we demonstrated that GFP cloned into the vectors was expressed as expected after chromosomal integration. This type of integration leads to the generation of duplicated sequences on either side of the integrated fragment^1^, which may serve as homology regions for further recombination that leads to the deletion of the integrated fragment, as pointed out previously^3^. To overcome this problem, integration vectors that do not produce repetitive genomic regions by employing double crossover recombination were developed^3^. Such stable replacements are suitable for long-term studies, although this comes at the expense of relatively low transformation efficiency. On the other hand, single crossover integration vectors show high transformation efficiency similar to that obtained by conventional plasmid introduction, even allowing for a small risk of producing further recombination. From these properties, single crossover vectors may be suitable for qualitative and quantitative experiments requiring high transformation efficiency such as screening using plasmid libraries. Practically, transformants obtained from the vectors developed in this study as well as pDUAL can be maintained in a medium lacking corresponding amino acids, thereby easily exerting selective pressure on the transformants.

One major concern associated with the utilization of the CRISPR/Cas9 system is the possibility of off-target effects, which involve the introduction of unintended mutations into genomic locations other than the intended target. In our study, we encountered several auxotrophic strains that retained the marker genes for the target, possibly as a result of off-target effects. Moreover, we observed an unexpected incorporation of a fragment of salmon DNA into the *arg3* ORF. The inadvertent integration of exogenous DNA into the genome of the host organism represents an additional potential issue when employing the CRISPR/Cas9 system. Previous studies have reported the insertion of salmon DNA fragments into the genome of fission yeast^20^, and similar occurrences have also been documented in other organisms^23, 24^. Hence, while CRISPR/Cas9 offers substantial potential for genome editing, it is imperative to diligently consider off-target effects and unintended insertions. Although the process of obtaining the *arg3-R25* allele was unexpected, the insertion of exogenous DNA at the 5’ region of the *arg3* ORF is of great advantage to this experimental system. This is because the insertion exerts selection pressure on the recombination region downstream of the inserted point, which is ideal for obtaining proper integrants that undergo arginine auxotrophy (Fig. 4b). Thus, we selected *arg3-R25* as the most suitable allele for targeting *arg3* using the pCArg3-series vectors.

In fission yeast research, *leu1*^+^ and *ura4*^+^ have been mainly used as selection markers^1^. The most commonly used *leu1* allele of the host is *leu1-32,* having a missense mutation in the *leu1* ORF^4^. On the other hand, the most common *ura4* marker used in the host strain is the complete deletion *ura4-D18*^18^. Therefore, *ura4-D18* cannot be used for single crossover recombination. Although vectors employing other selection marker genes have been gradually developed, we still have limited options for chromosomal integration of a transgene. The vectors developed in this study will serve as a valuable option for heterologous gene expression from the chromosome in combination with most of the previously reported markers. In this context, these vectors will contribute considerably to experiments requiring the expression of multiple transgenes.

## Supplementary Information

Supplementary Figures.

Supplementary Information.

## Data Availability

All data generated or analyzed during this study are included in this published article and its Supplementary Information files.

## Abbreviations

PCR: Polymerase chain reaction
ORF: Open reading frame
sgRNA: Single-guided RNA
GFP: Green fluorescent protein
CFP: Cyan fluorescent protein
YFP: Yellow fluorescent protein
