# Supplementary figures and images for: A set of vectors and strains for chromosomal integration in fission yeast
Source: Sci Rep. 2023 Jun 8;13:9295. doi: 10.1038/s41598-023-36267-1 (PMC10250367; doi:10.1038/s41598-023-36267-1)

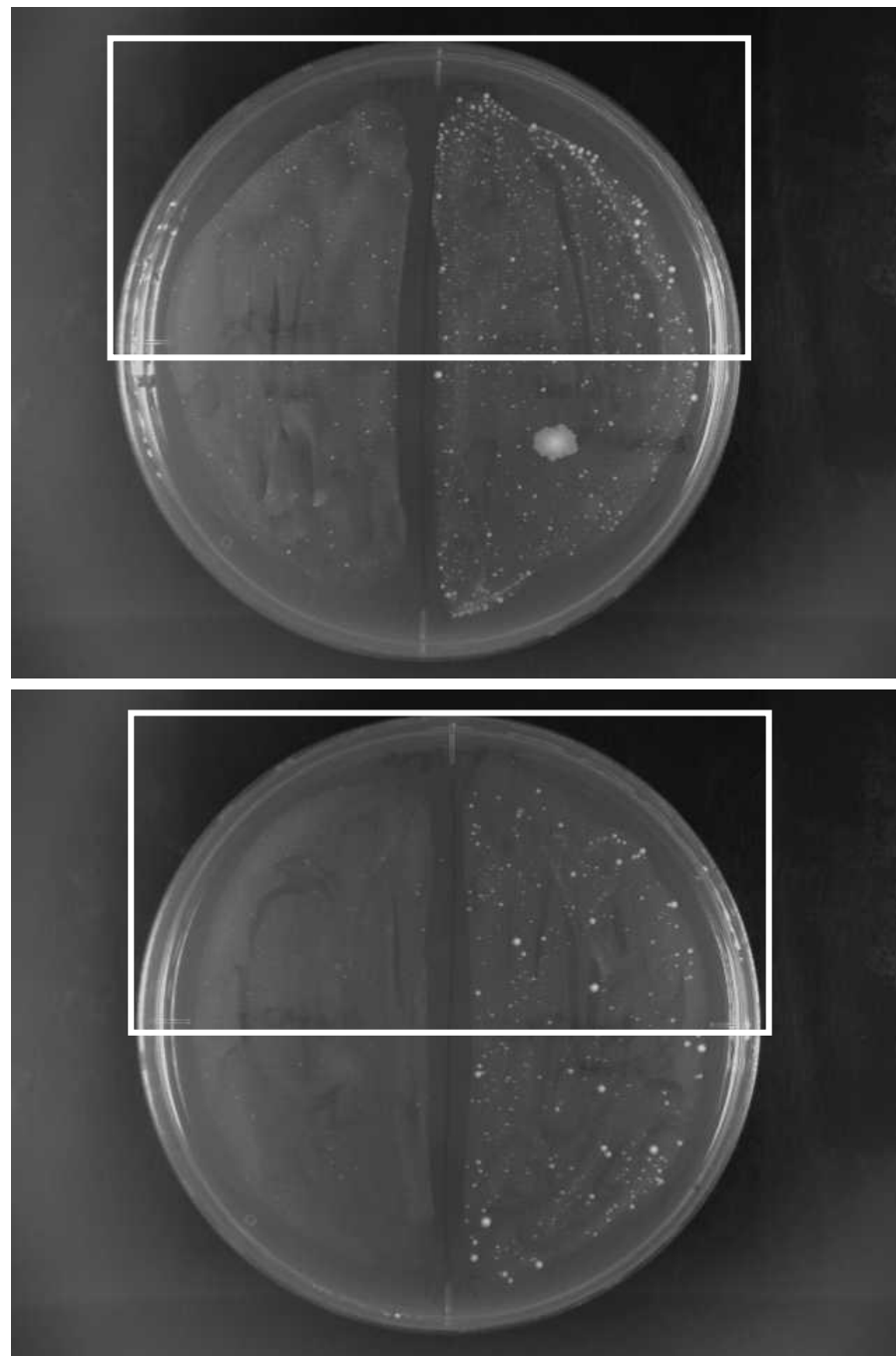

**(Fig. 5A)**

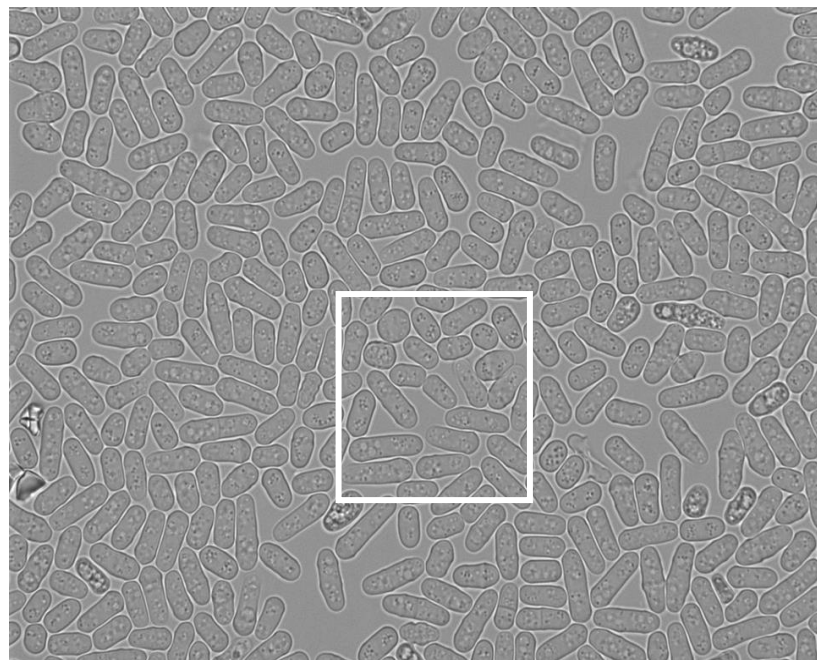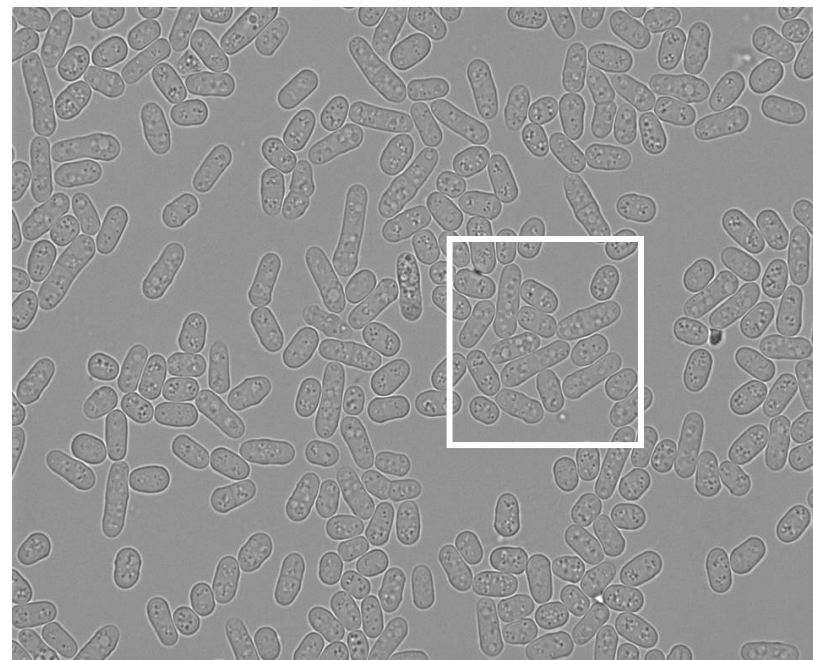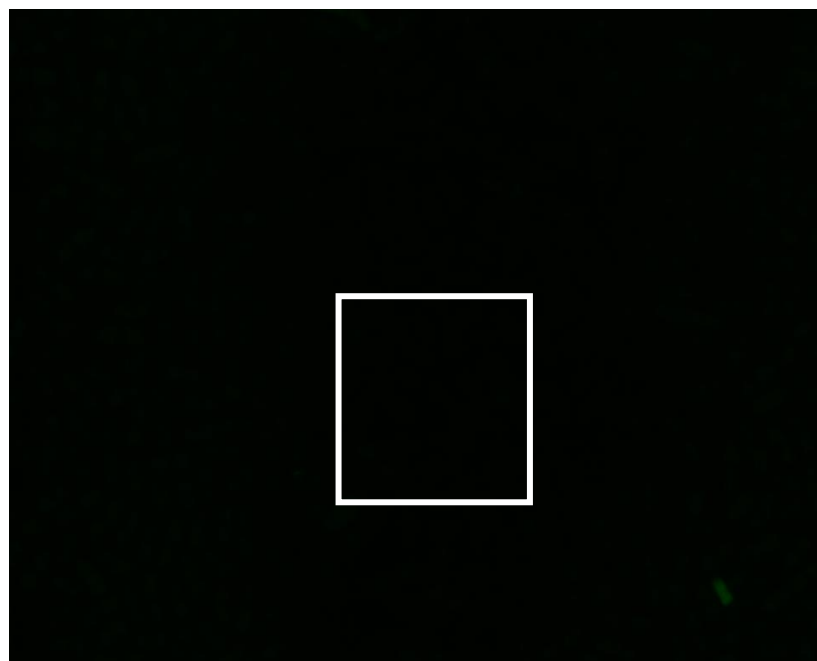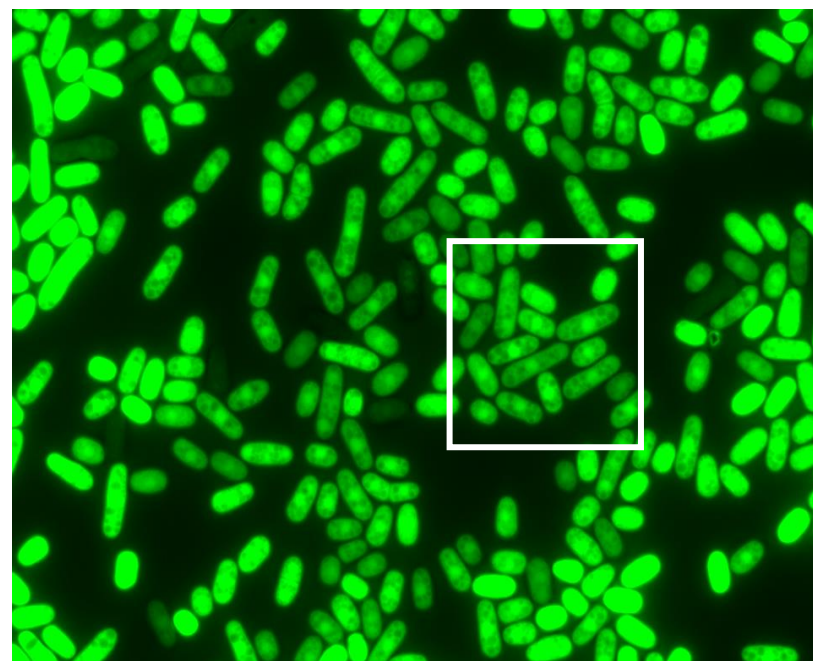

**(Fig. 5B, left)**

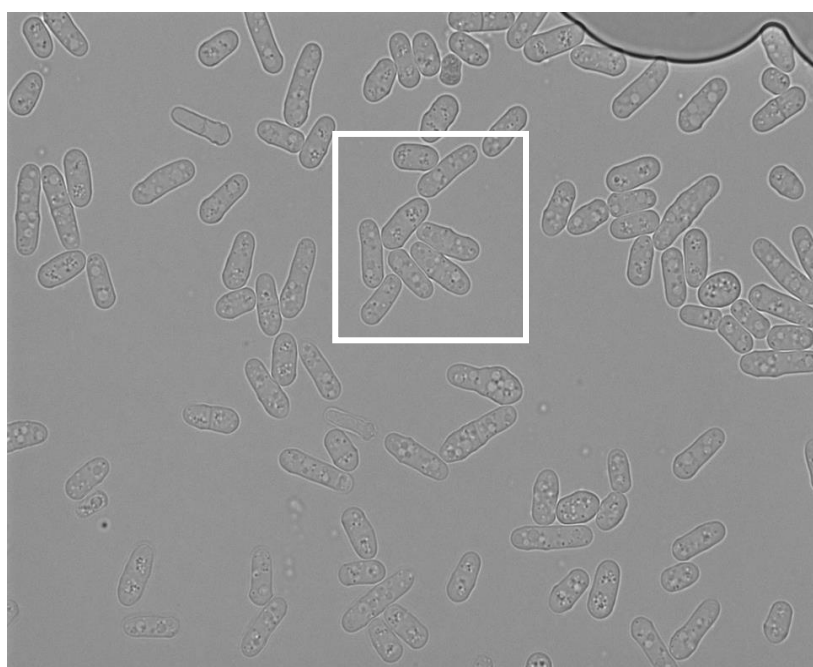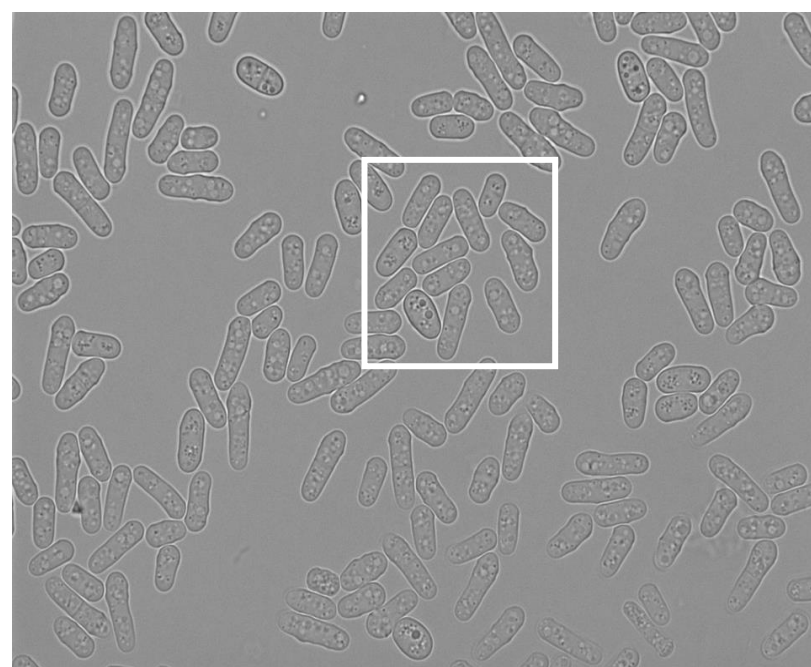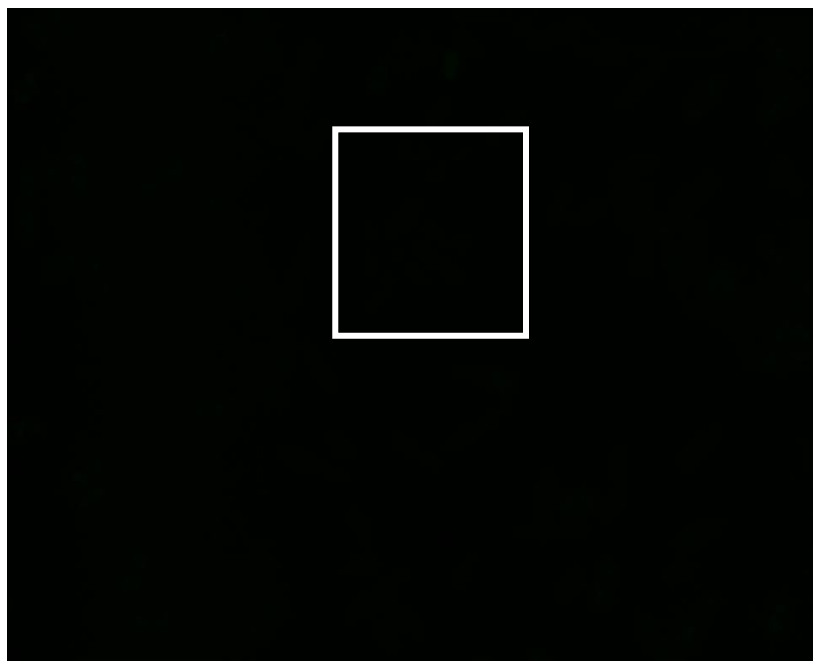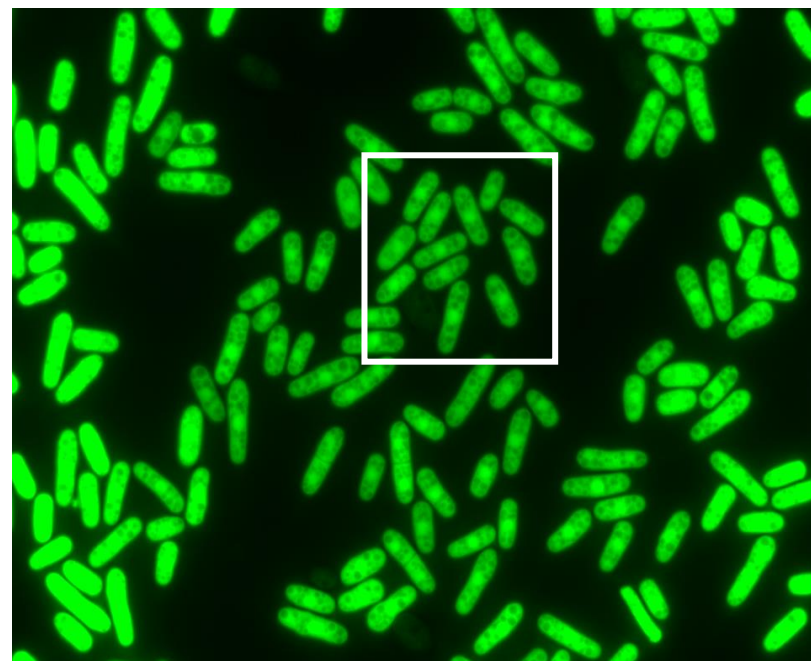

**(Fig. 5B, right)**

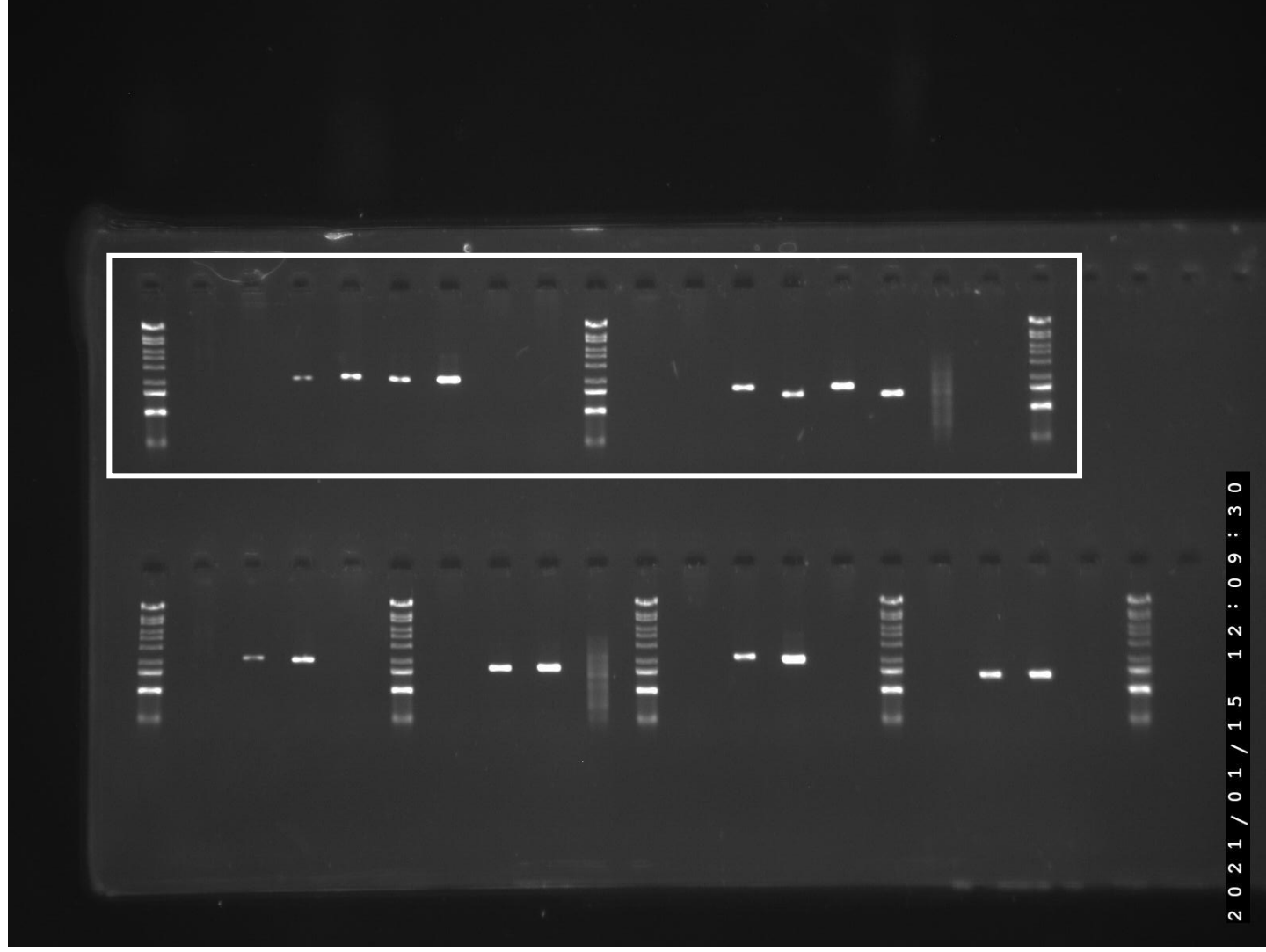

(Fig. 5C)

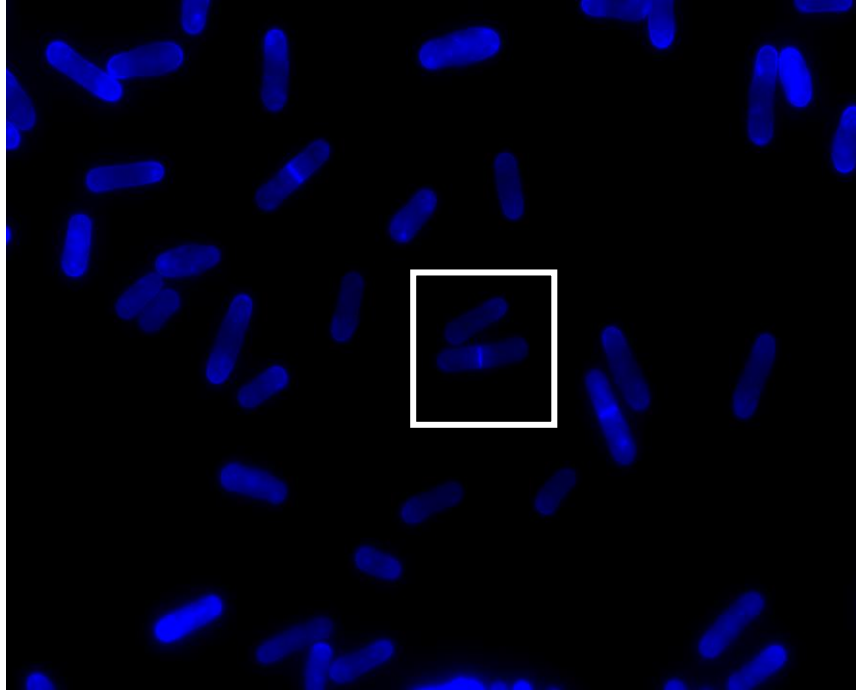

**Uvi15-CFP  
(pCLys1)**

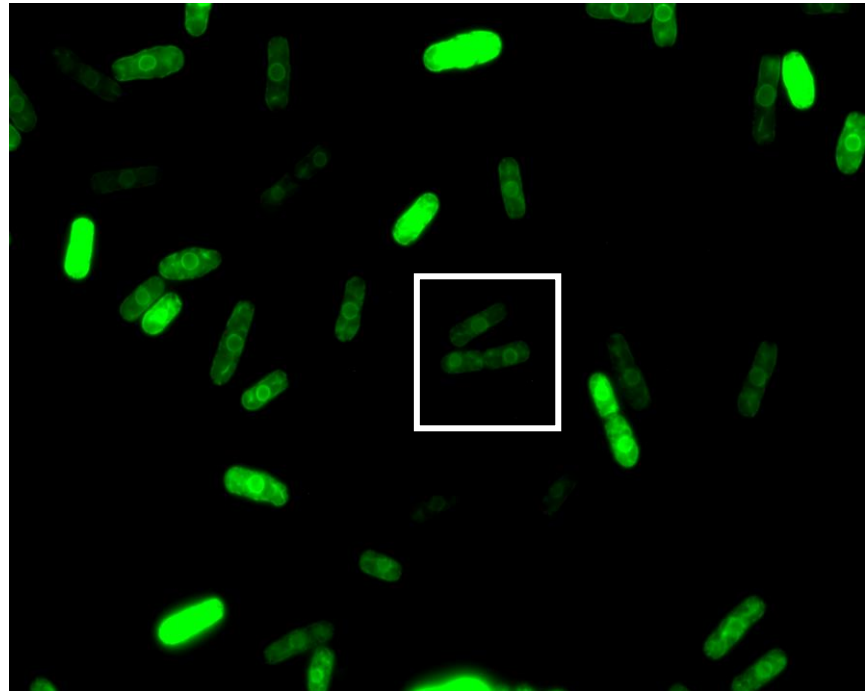

**Gpi16-YFP  
(pDUAL)**

**Gar2-mCherry  
(pCArg3)**

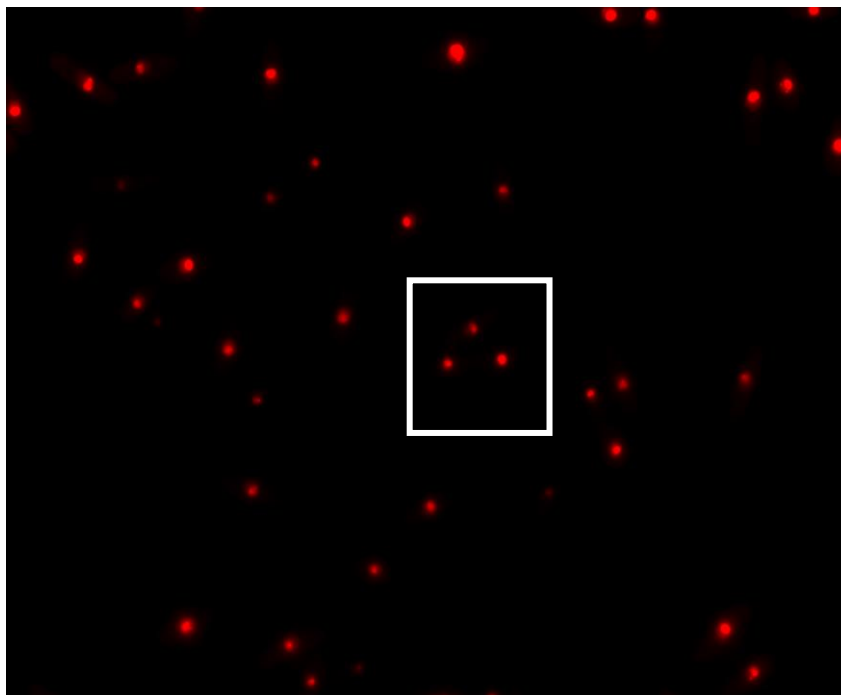

**(Fig. 5D)**

Supplement: Supplementary file 1 — Supplementary Figures. [file 41598_2023_36267_MOESM1_ESM.pdf]

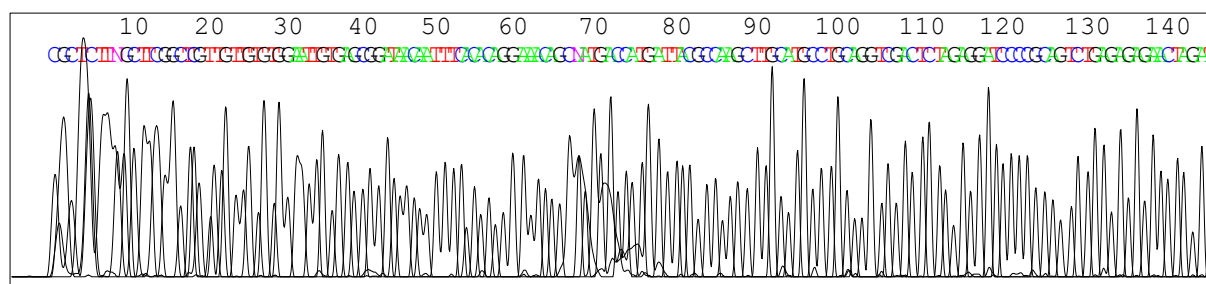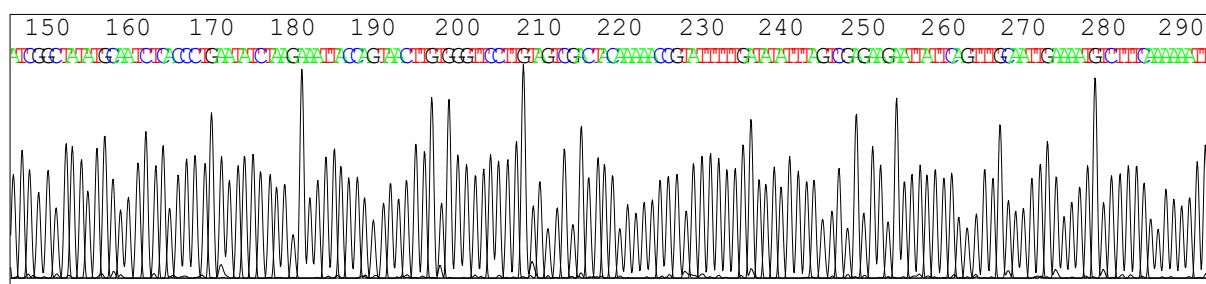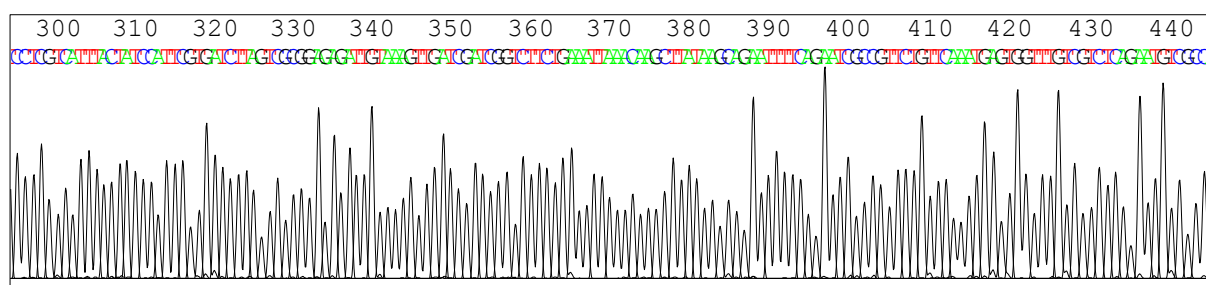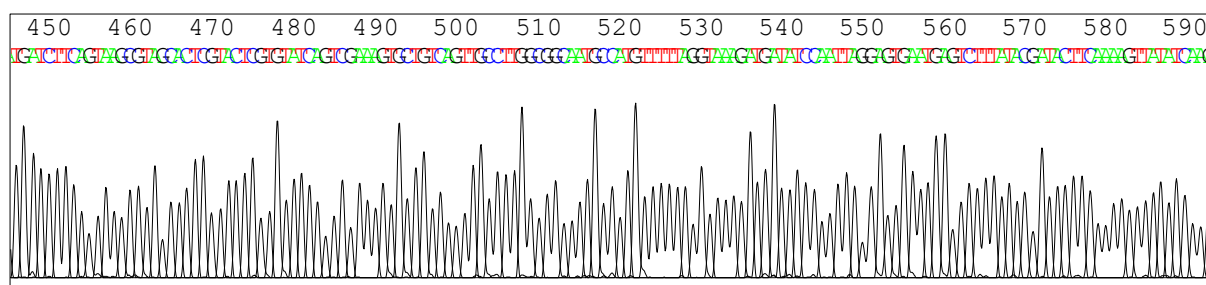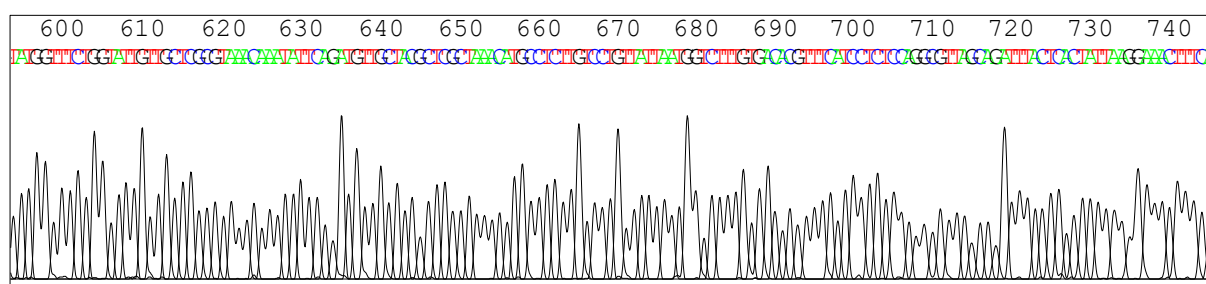

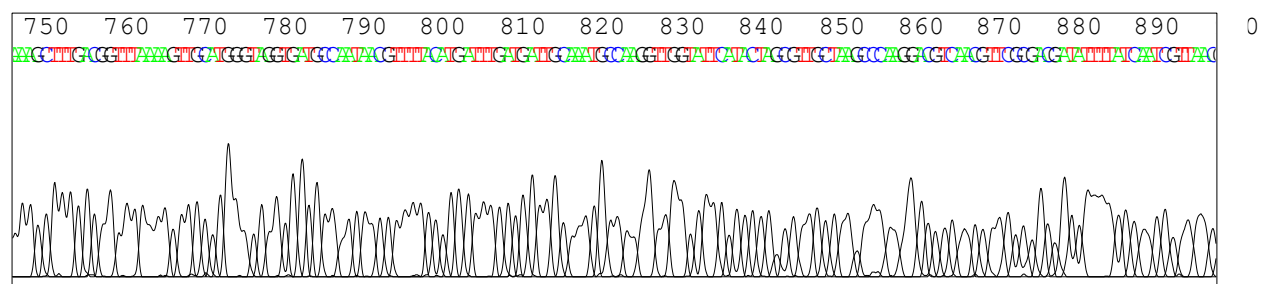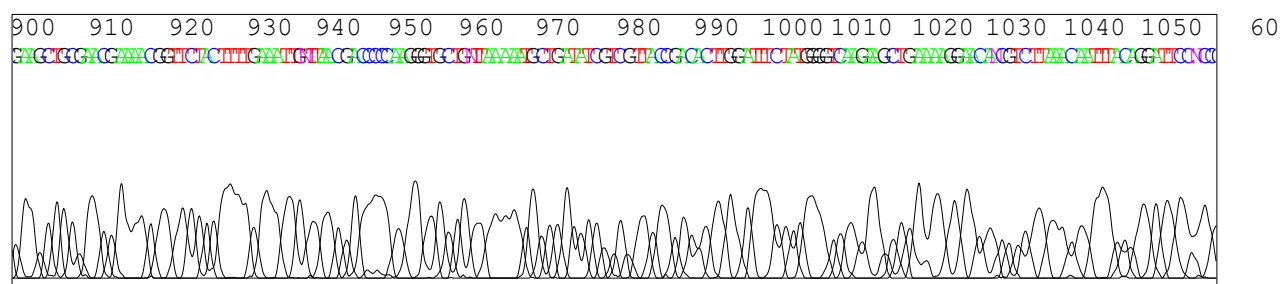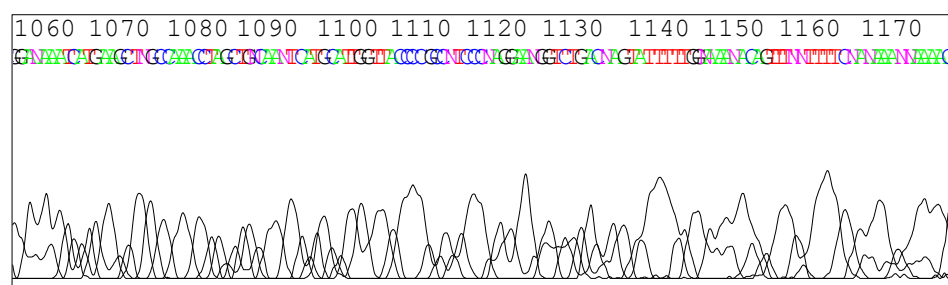

Supplement: Supplementary file 2 — Supplementary Information. [file 41598_2023_36267_MOESM2_ESM.zip › arg3-1_R12_Rv.pdf]

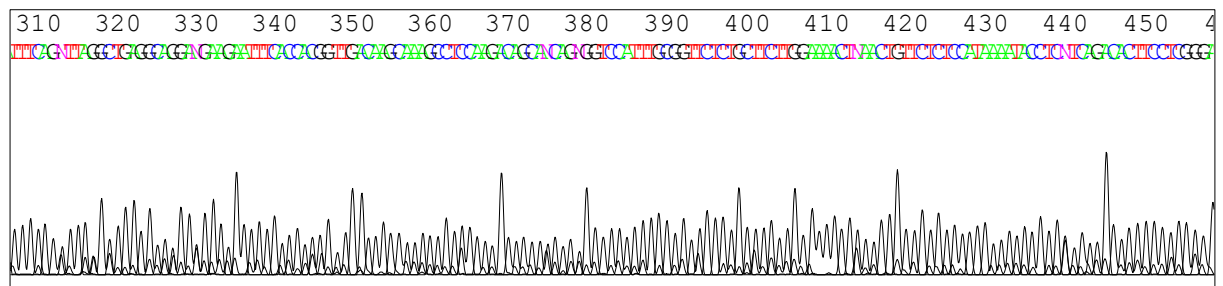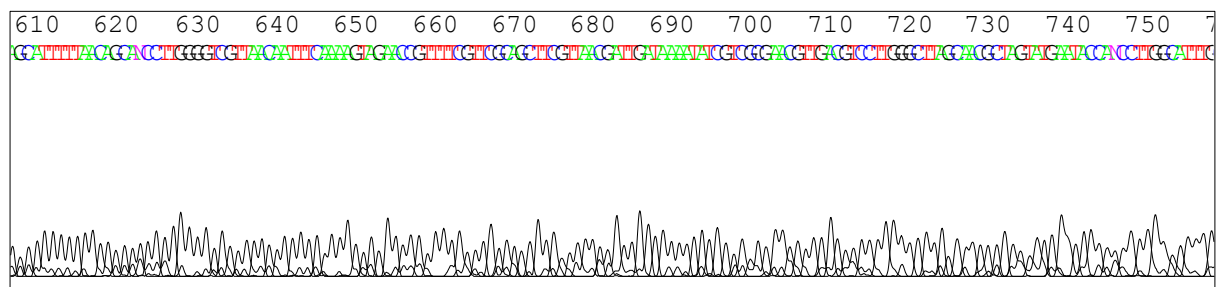

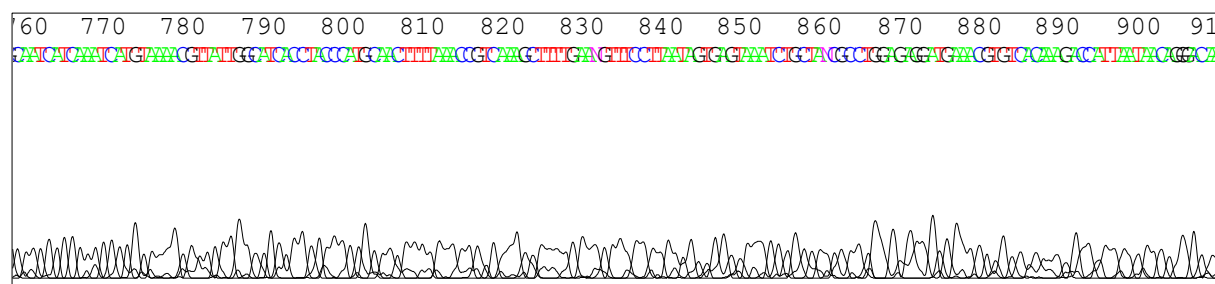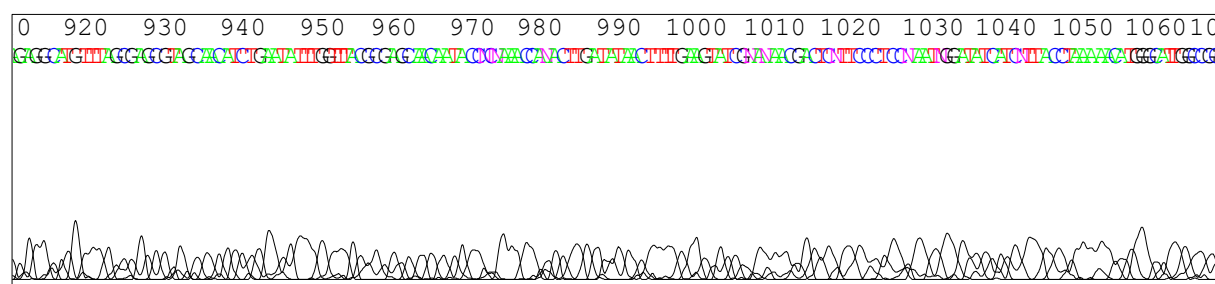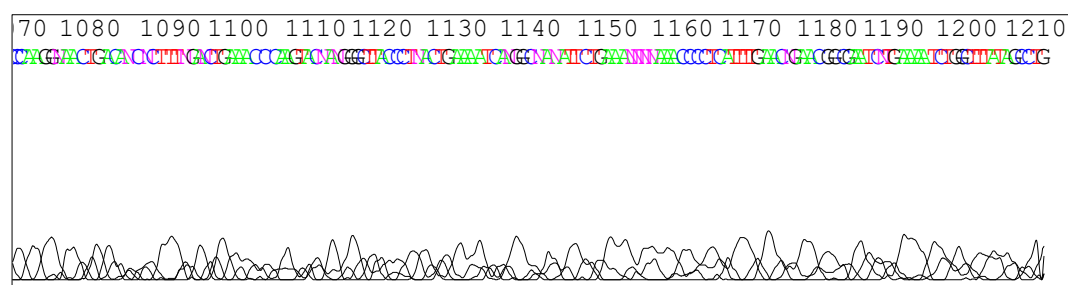

Supplement: Supplementary file 2 — Supplementary Information. [file 41598_2023_36267_MOESM2_ESM.zip › arg3-1_R13_Fw.pdf]

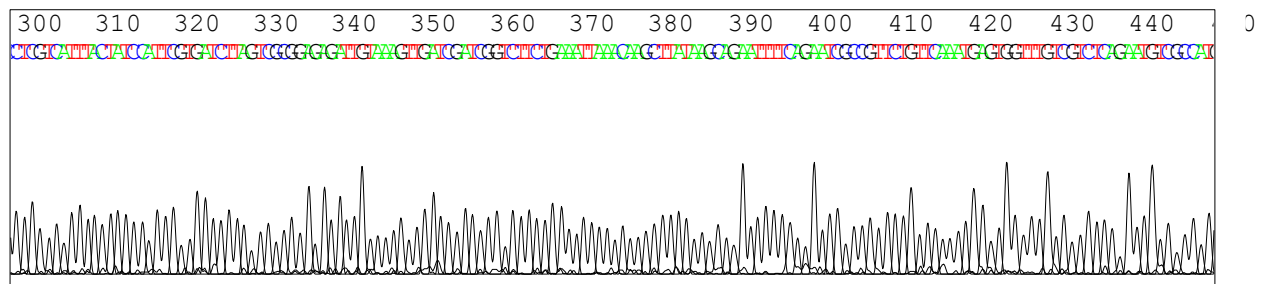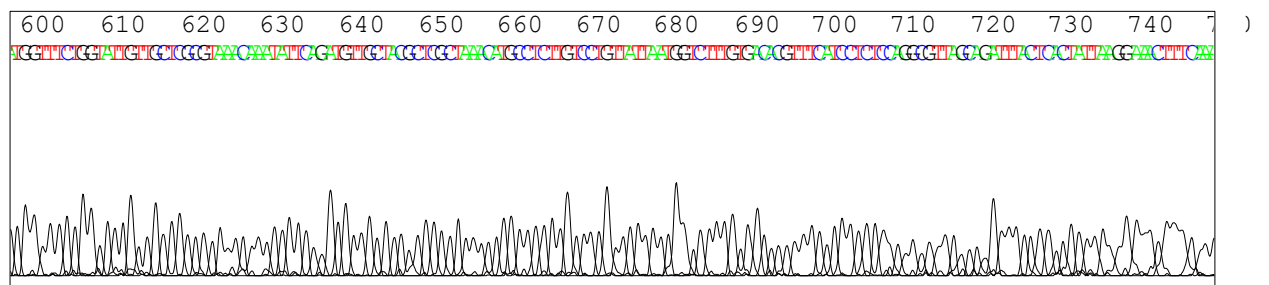

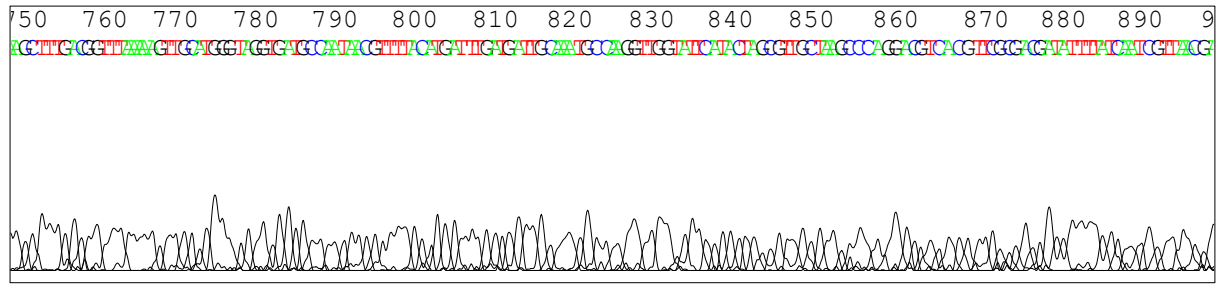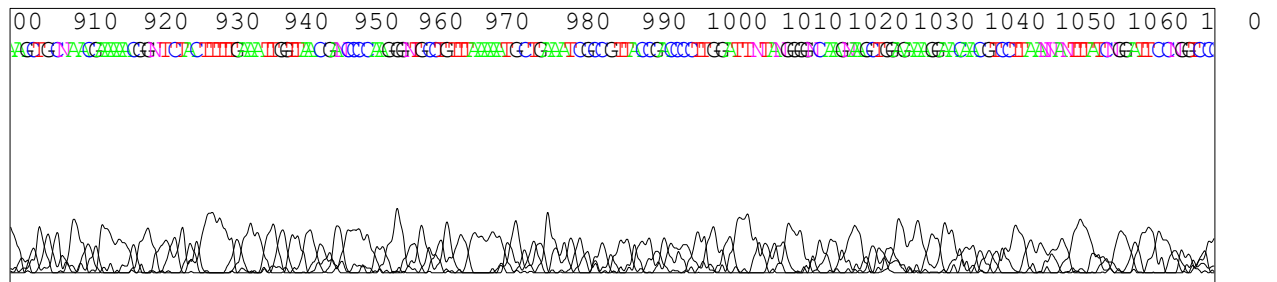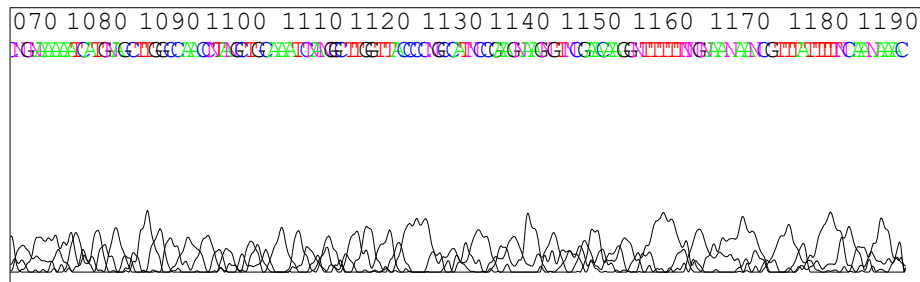

Supplement: Supplementary file 2 — Supplementary Information. [file 41598_2023_36267_MOESM2_ESM.zip › arg3-1_R13_Rv.pdf]

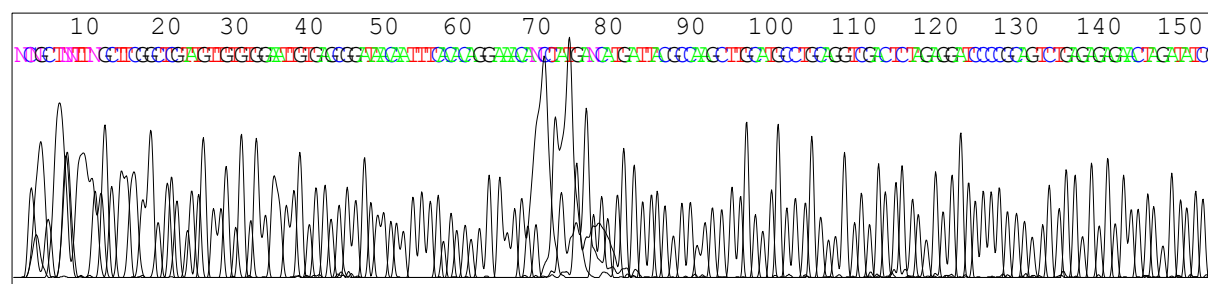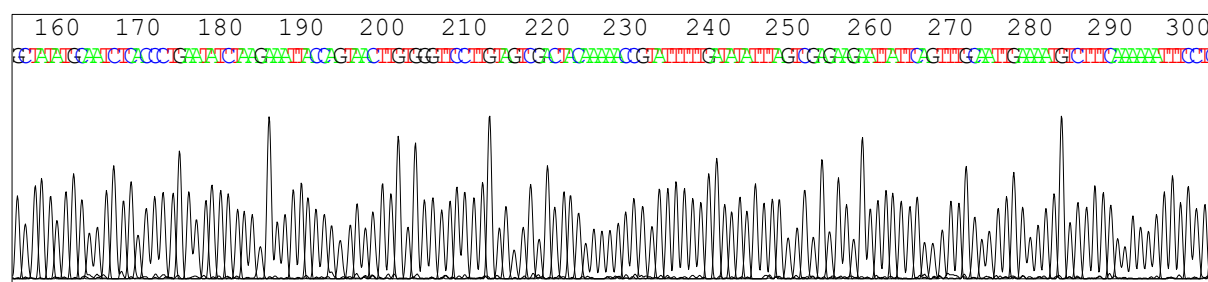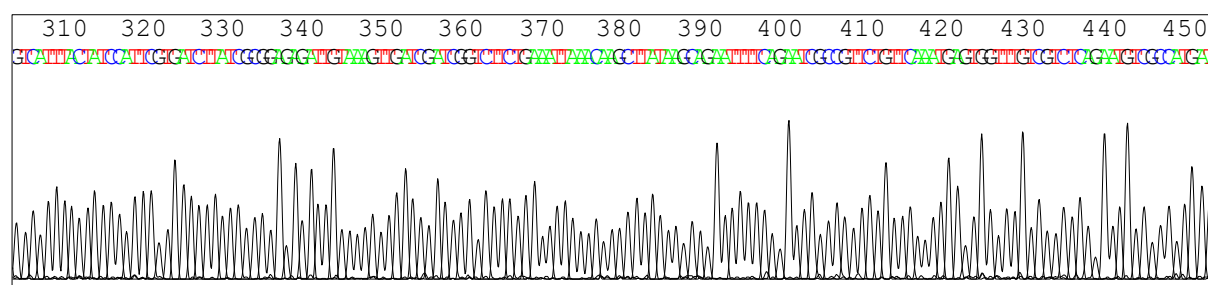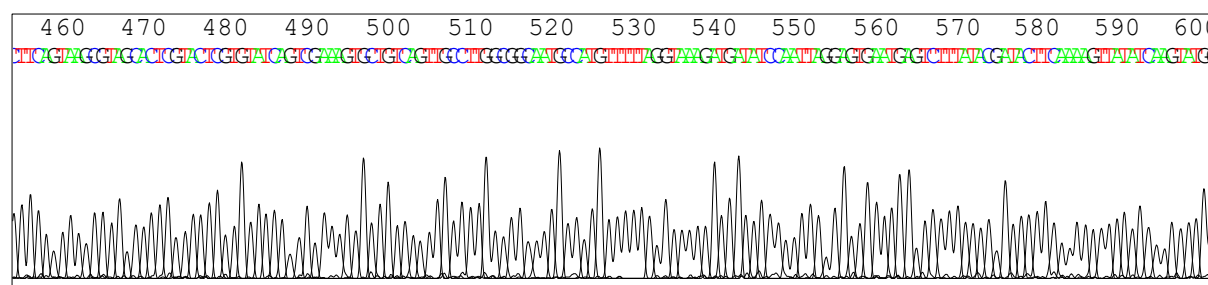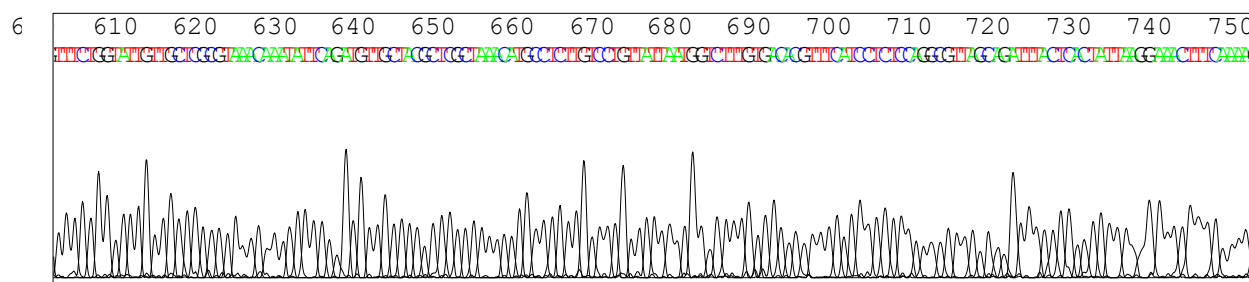

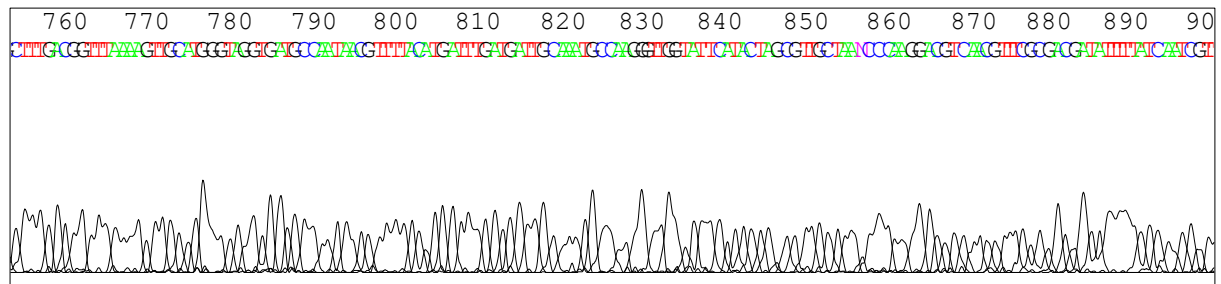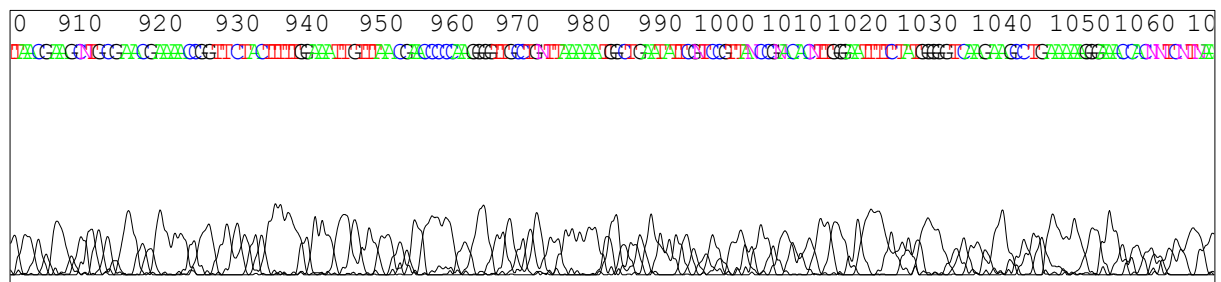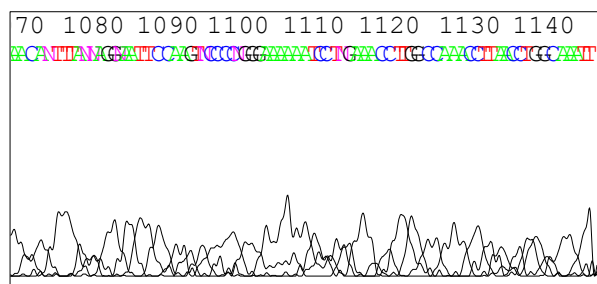

Supplement: Supplementary file 2 — Supplementary Information. [file 41598_2023_36267_MOESM2_ESM.zip › arg3-2_R21_Rv.pdf]

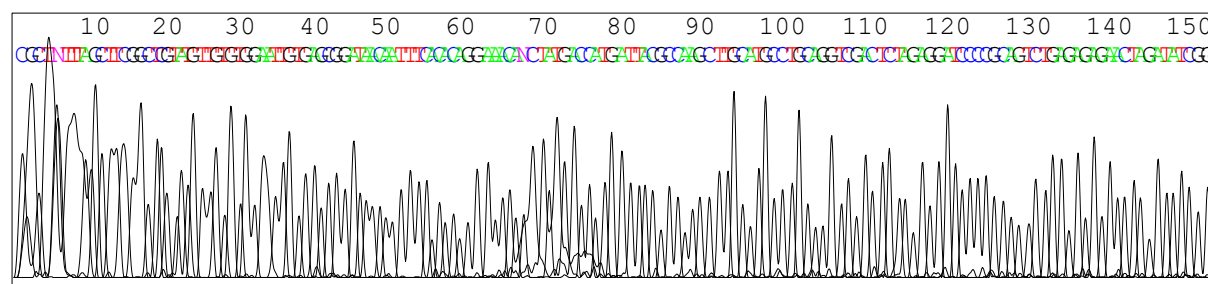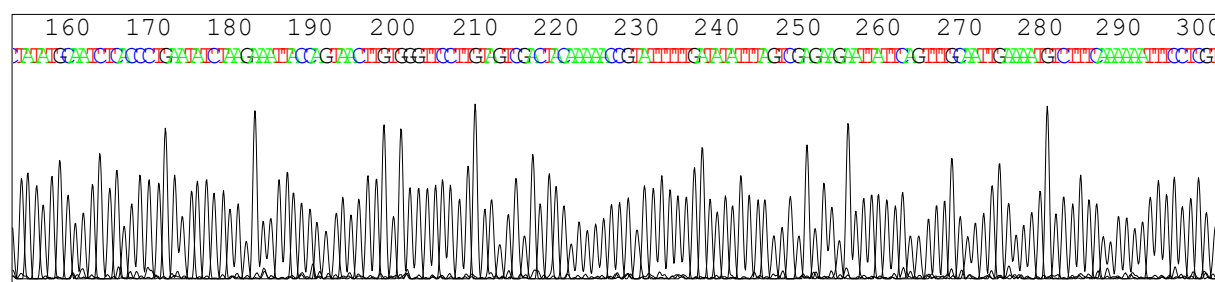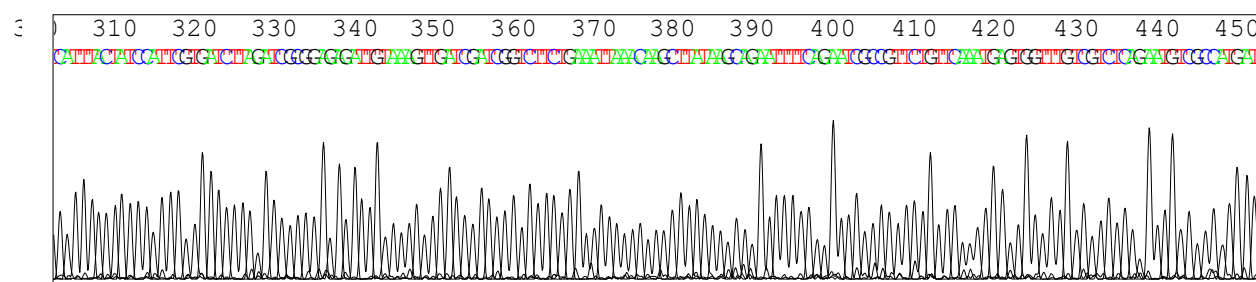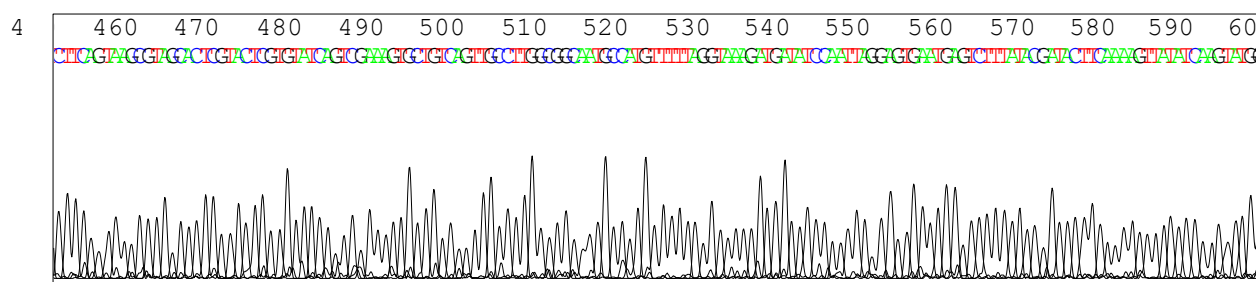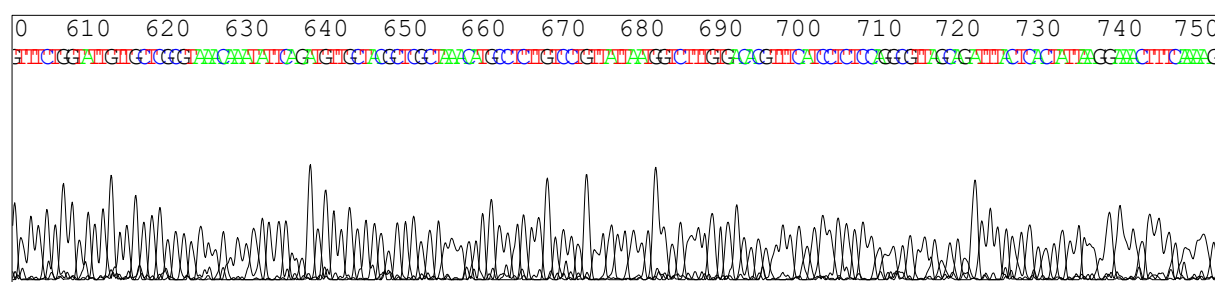

Supplement: Supplementary file 2 — Supplementary Information. [file 41598_2023_36267_MOESM2_ESM.zip › arg3-2_R26_Rv.pdf]

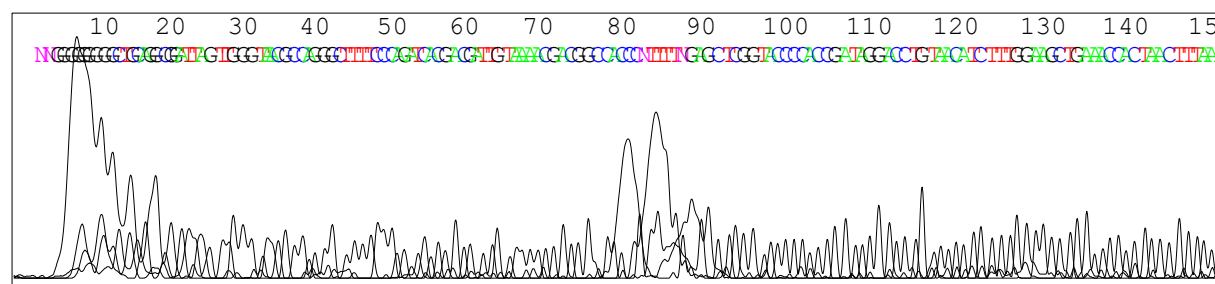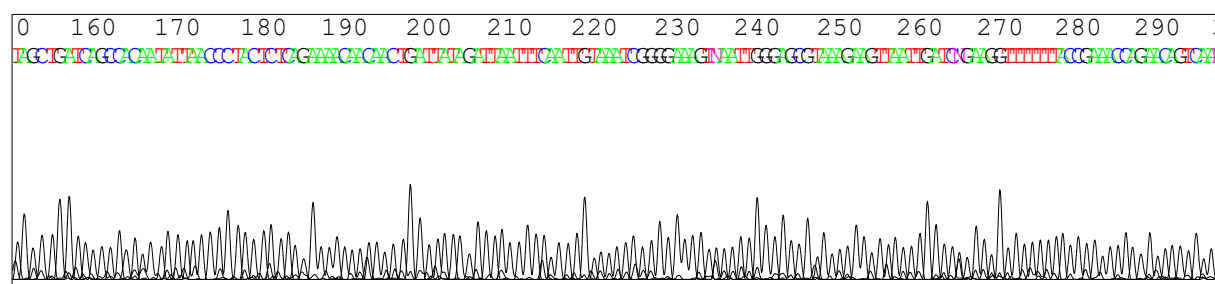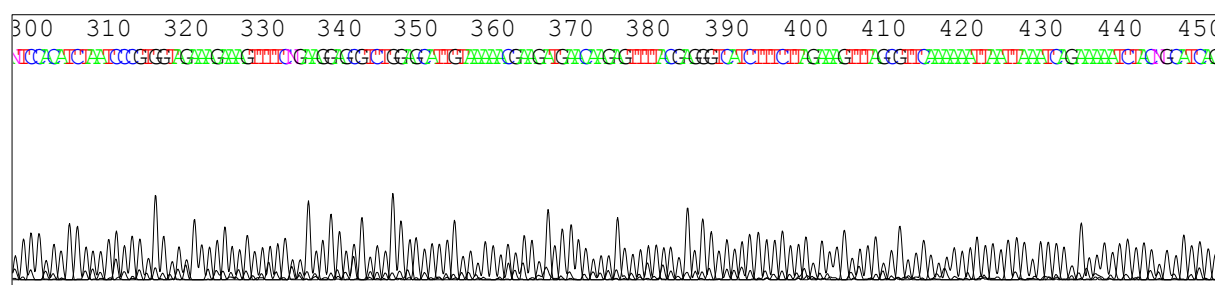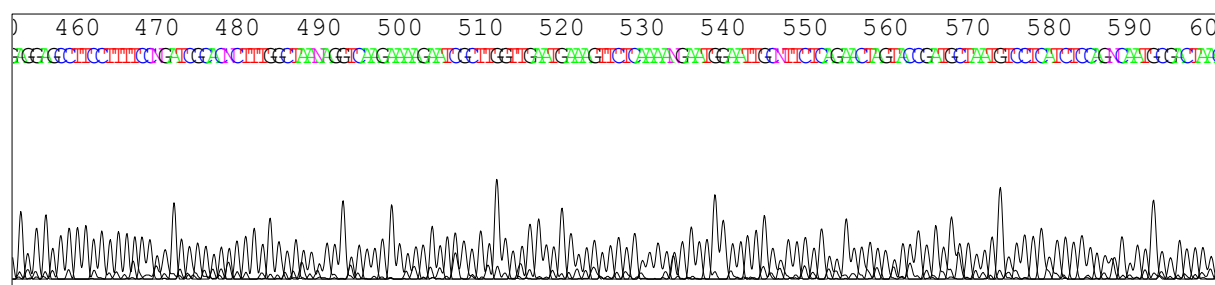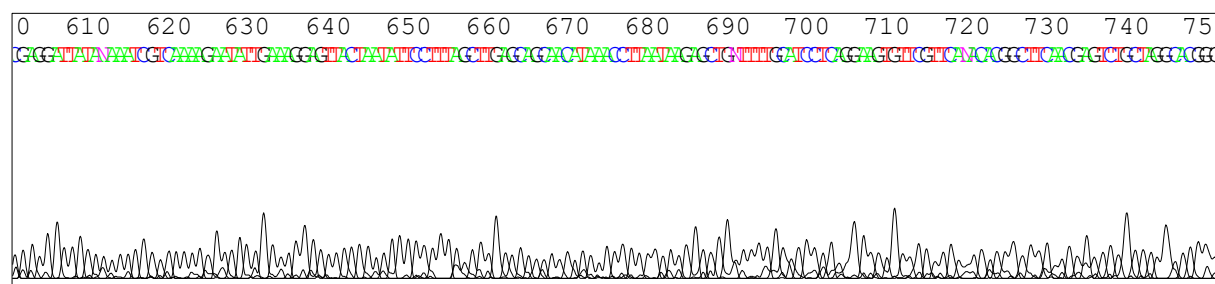

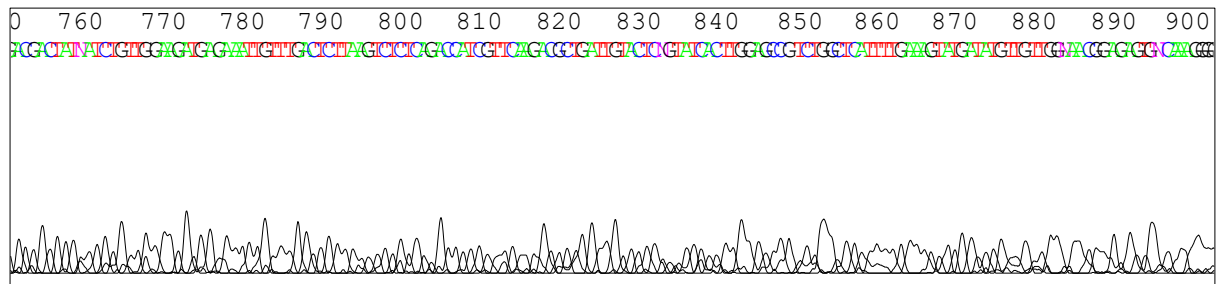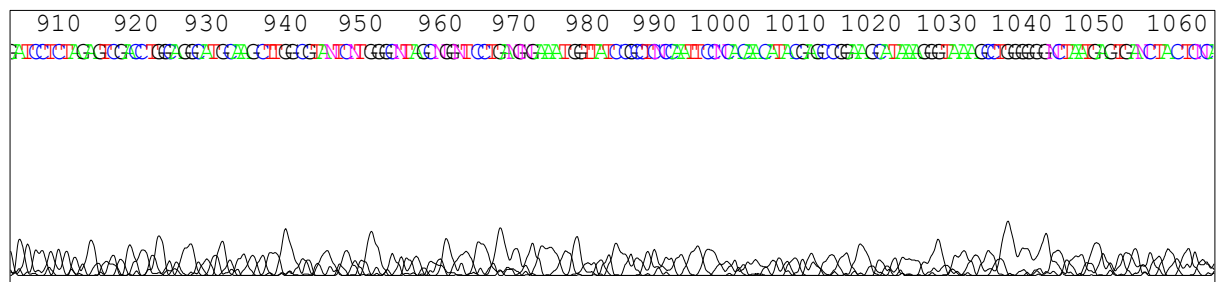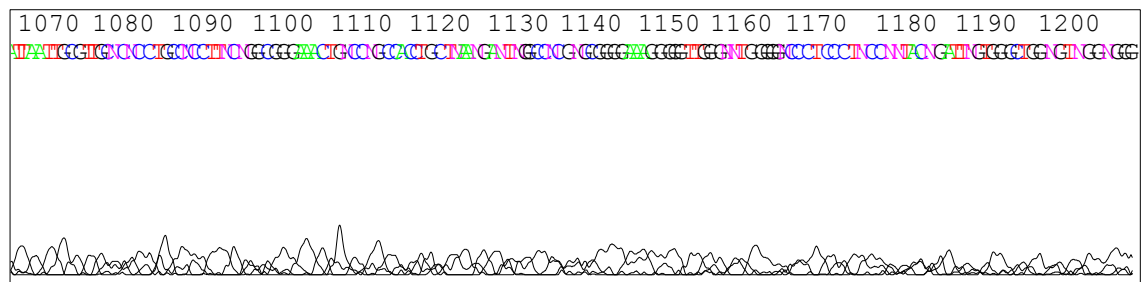

Supplement: Supplementary file 2 — Supplementary Information. [file 41598_2023_36267_MOESM2_ESM.zip › lys1-2_K22_Fw.pdf]

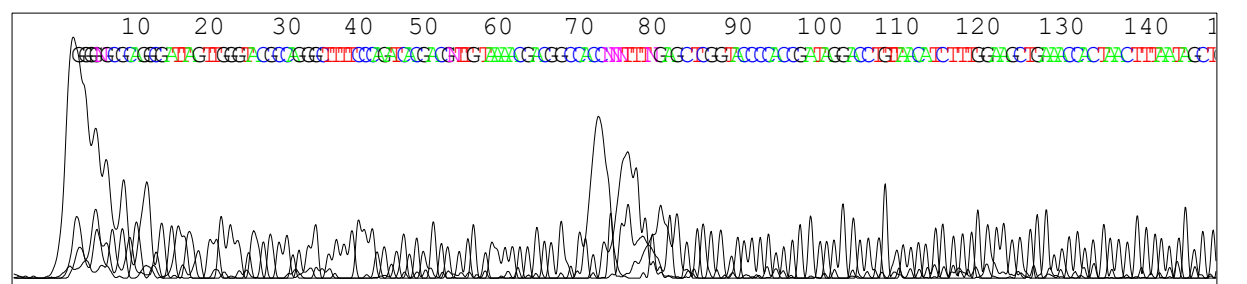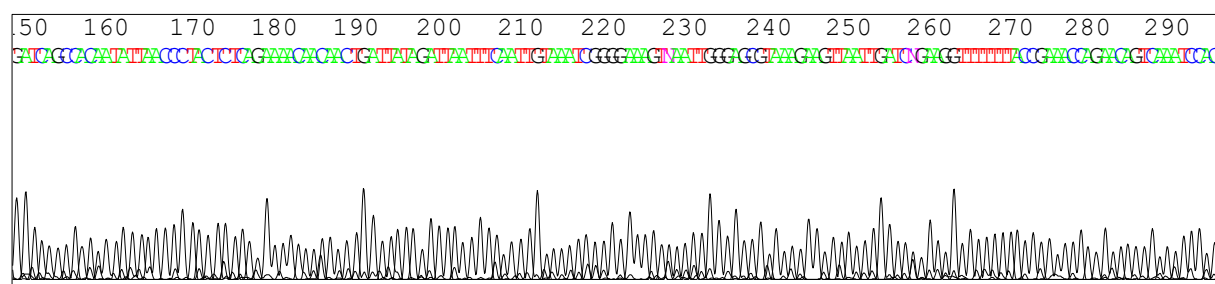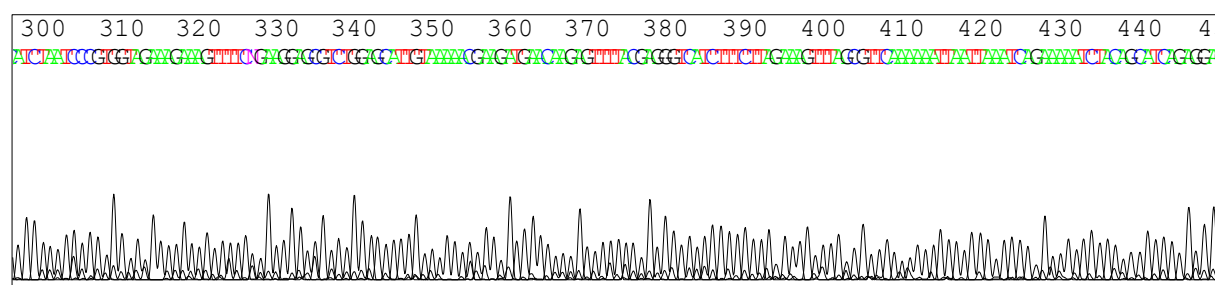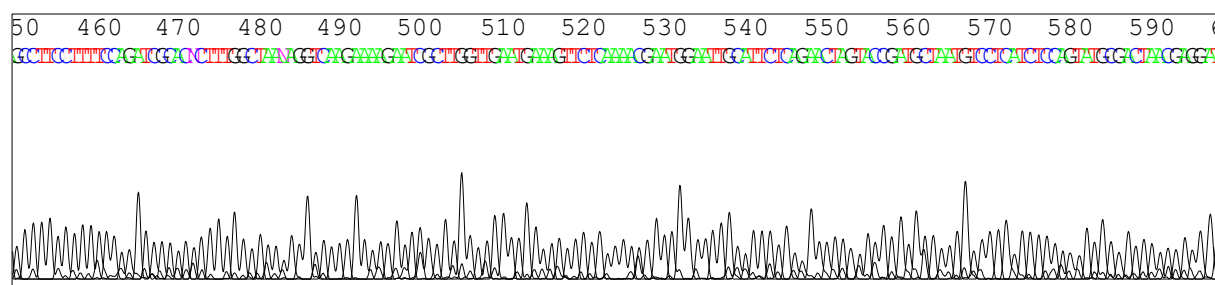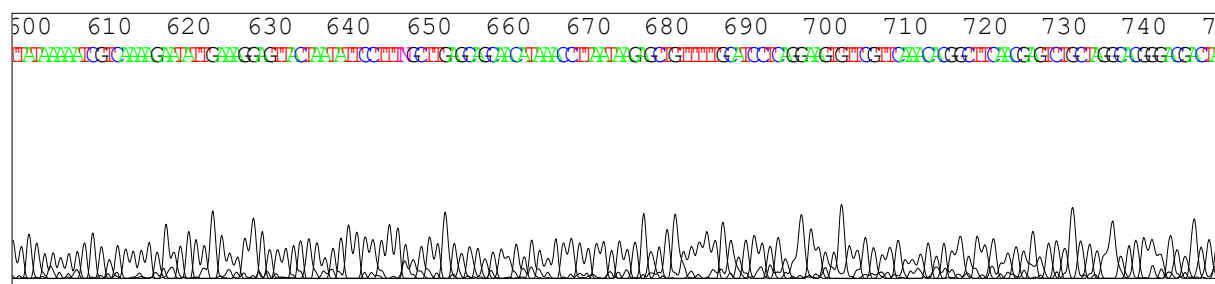

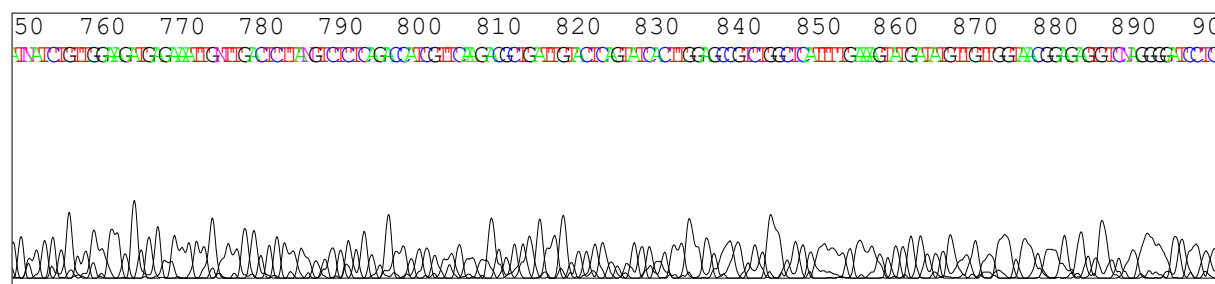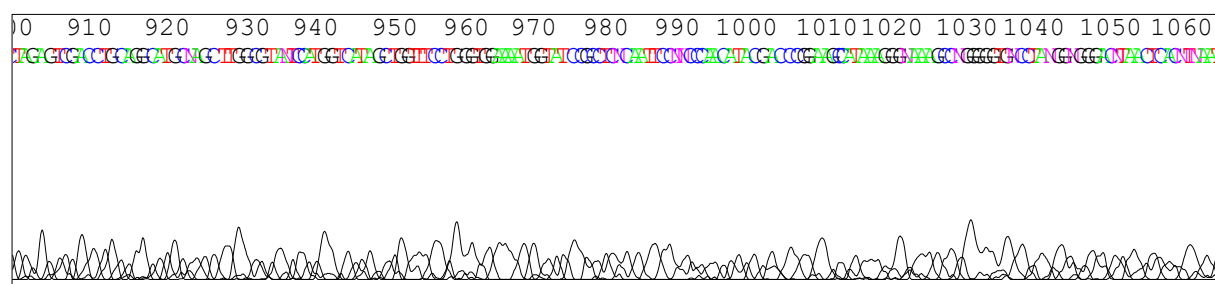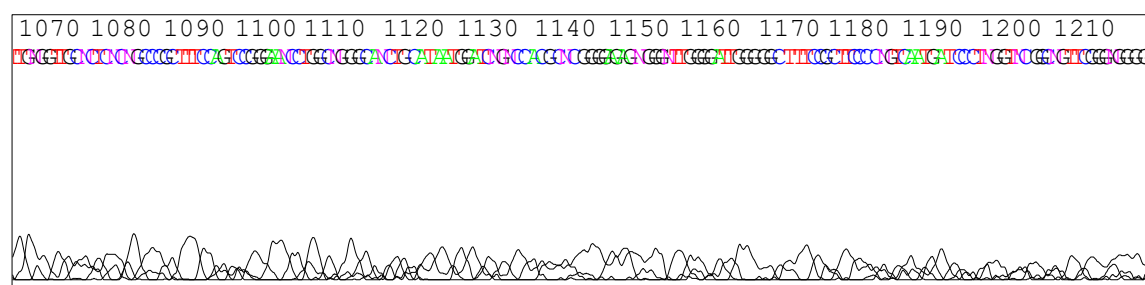

Supplement: Supplementary file 2 — Supplementary Information. [file 41598_2023_36267_MOESM2_ESM.zip › lys1-2_K23_Fw.pdf]

10 20 30 40 50 60 70 80 90 100 110 120 130 140 150

GGTATGTTGGTCTGGGATGGGAGACATTACAGTACGATAGAGCTGATCTGAGTCTTGGATCCCTGCTCTACAA

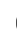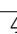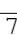

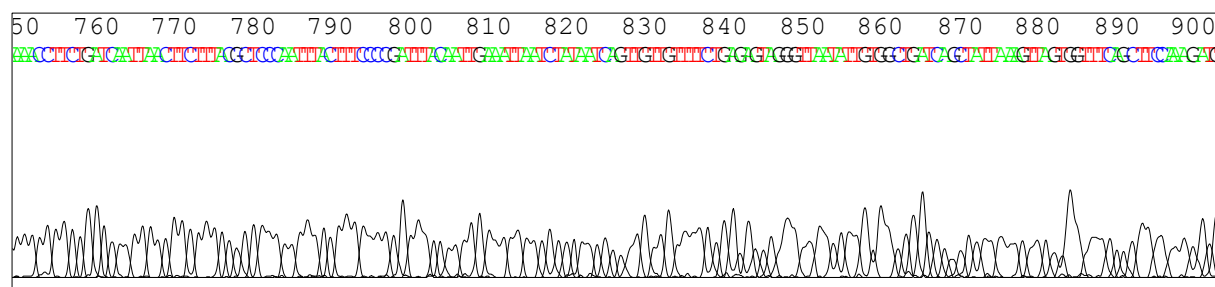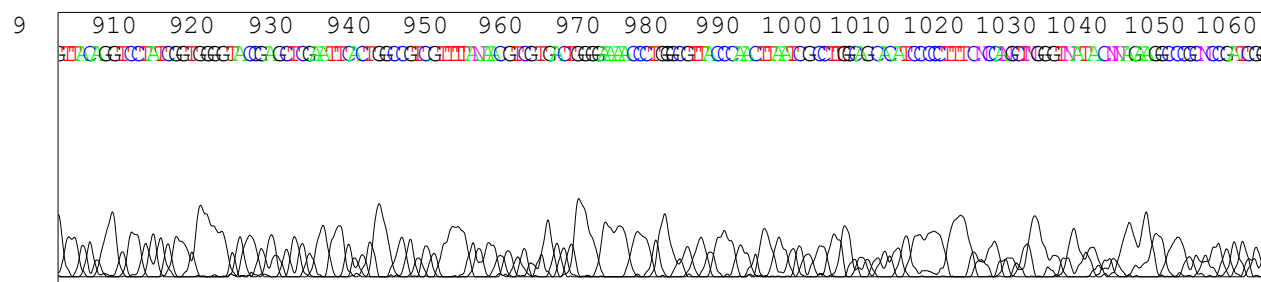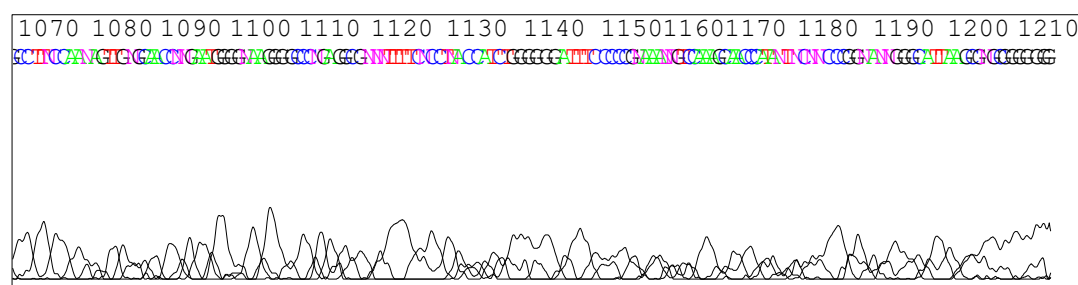

Supplement: Supplementary file 2 — Supplementary Information. [file 41598_2023_36267_MOESM2_ESM.zip › lys1-2_K24_Rv.pdf]
