# Supplementary material for: A set of vectors and strains for chromosomal integration in fission yeast
Source: Sci Rep. 2023 Jun 8;13:9295. doi: 10.1038/s41598-023-36267-1 (PMC10250367; doi:10.1038/s41598-023-36267-1)

File: C:/Users/gifus/Box/Per_020474_Akihisa Matsuyama/Paper_˜•¶/New host-vector system/Raw data/Sequence results
Run ended: 5:19PM Mon,Dec 23, 2019
(page 1)

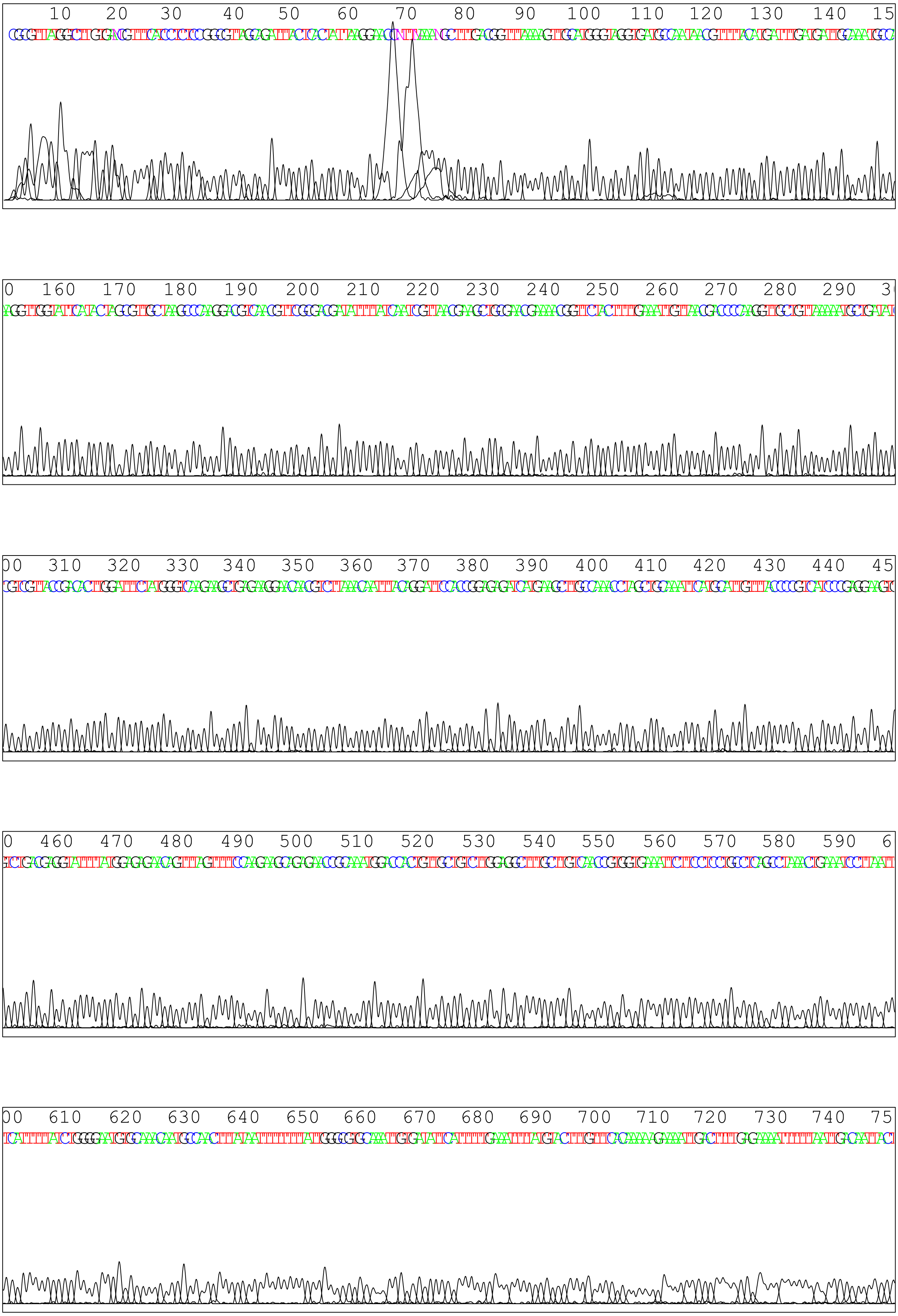

File: C:/Users/gifus/Box/Per_020474_Akihisa Matsuyama/Paper_˜•¶/New host-vector system/Raw data/Sequence results
Run ended: 5:19PM Mon,Dec 23, 2019
(page 2)

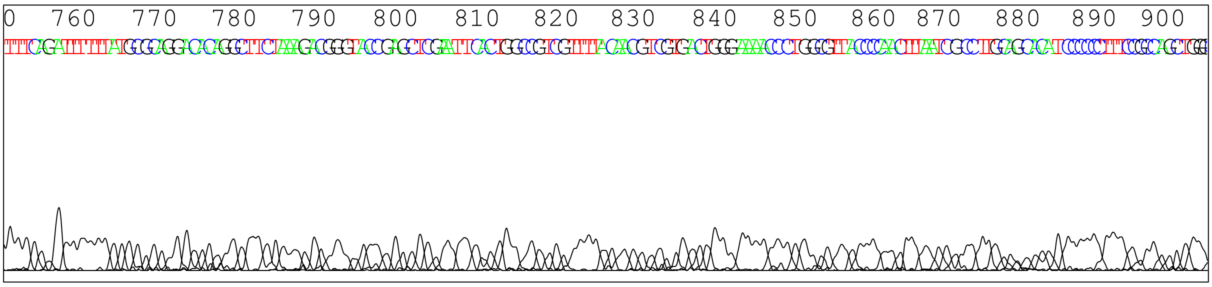

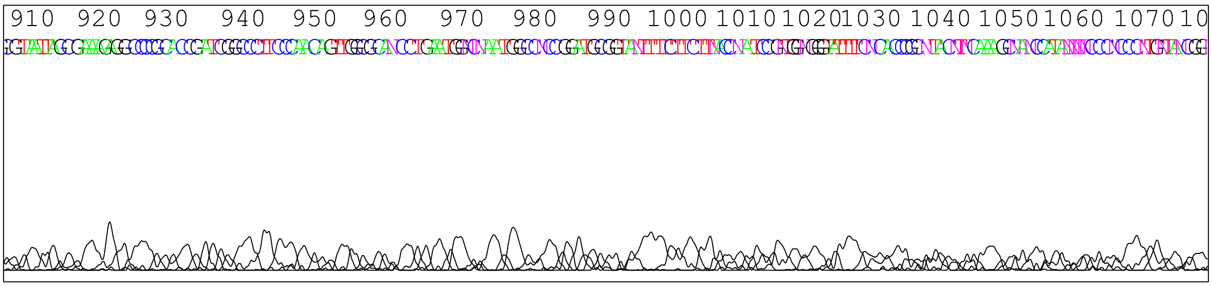

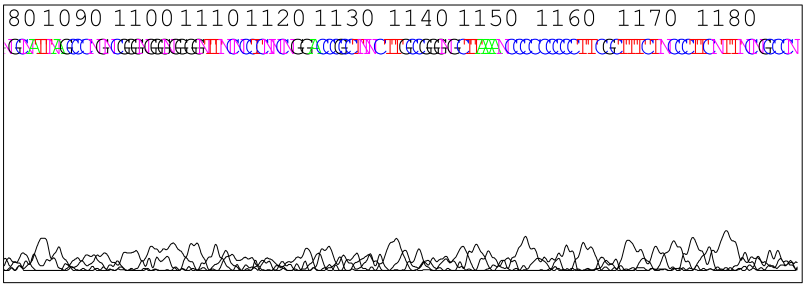

Supplement: Supplementary file 2 — Supplementary Information. [file 41598_2023_36267_MOESM2_ESM.zip › arg3-1_R12_inside.pdf]
